# Molecular structures reveal the origin of spectral variation in cryptophyte light harvesting antenna proteins

**DOI:** 10.1002/pro.4586

**Published:** 2023-02-24

**Authors:** Katharine A. Michie, Stephen J. Harrop, Harry W. Rathbone, Krystyna E. Wilk, Chang Ying Teng, Kerstin Hoef‐Emden, Roger G. Hiller, Beverley R. Green, Paul M. G. Curmi

**Affiliations:** ^1^ School of Physics The University of New South Wales Sydney New South Wales Australia; ^2^ School of Biotechnology and Biomolecular Sciences The University of New South Wales Sydney New South Wales Australia; ^3^ Mark Wainwright Analytical Centre University of New South Wales Sydney New South Wales Australia; ^4^ MX Beamlines, Australian Synchrotron Clayton Victoria Australia; ^5^ Department of Botany University of British Columbia Vancouver Canada; ^6^ Botanical Institute University of Cologne Cologne Germany; ^7^ Department of Biological Sciences Macquarie University Sydney New South Wales Australia

**Keywords:** cryptophyte, evolution, light harvesting protein, phycobiliprotein, x‐ray crystallography

## Abstract

In addition to their membrane‐bound chlorophyll a/c light‐harvesting antenna, the cryptophyte algae have evolved a unique phycobiliprotein antenna system located in the thylakoid lumen. The basic unit of this antenna consists of two copies of an αβ protomer where the α and β subunits scaffold different combinations of a limited number of linear tetrapyrrole chromophores. While the β subunit is highly conserved, encoded by a single plastid gene, the nuclear‐encoded α subunits have evolved diversified multigene families. It is still unclear how this sequence diversity results in the spectral diversity of the mature proteins. By careful examination of three newly determined crystal structures in comparison with three previously obtained, we show how the α subunit amino acid sequences control chromophore conformations and hence spectral properties even when the chromophores are identical. Previously we have shown that α subunits control the quaternary structure of the mature αβ.αβ complex (either open or closed), however, each species appeared to only harbor a single quaternary form. Here we show that species of the *Hemiselmis* genus contain expressed α subunit genes that encode both distinct quaternary structures. Finally, we have discovered a common single‐copy gene (expressed into protein) consisting of tandem copies of a small α subunit that could potentially scaffold pairs of light harvesting units. Together, our results show how the diversity of the multigene α subunit family produces a range of mature cryptophyte antenna proteins with differing spectral properties, and the potential for minor forms that could contribute to acclimation to varying light regimes.

## Abstract 2

PDB Code(s): 7T7U, 7T8S and 7T89;

## 1 INTRODUCTION

Around a billion years ago, the ancestor of the cryptophyte algae engulfed a unicellular red alga which became an endosymbiont. Over time, most of the red algal cellular machinery was lost, leaving behind its plastid and a remnant of its nucleus (the nucleomorph), although many nuclear genes were integrated into the host's nuclear genome (Archibald, 2020; Zimorski et al., 2014). What distinguishes the cryptophyte lineage from other algae with acquired red algal plastids is that the cryptophytes evolved a unique light‐harvesting antenna complex derived from two relict parts of the red algal phycobilisome (Archibald, 2020; Rathbone et al., 2021), which has been completely dismantled.

The red algal phycobilisome is a large, complex multisubunit structure made up of rods assembled from red algal phycobiliproteins, that covalently bind linear tetrapyrroles (phycobilins) rather than chlorophylls (Ma et al., 2020; Zhang et al., 2017). The basic unit of a rod is a hexameric (αβ)_3_ disk (Schirmer et al., 1985) where α and β are related proteins of the globin family each binding several phycobilins (Apt et al., 1995). These disks stack together to form the rod structures. Phycobilisomes are the major light‐harvesting antenna of red algae, and the only antenna in cyanobacteria and glaucophyte algae.

The first high‐resolution crystal structure of a cryptophyte phycobiliprotein (phycoerythrin 545, PE545, from *Rhodomonas* CS24) showed that it was made up of two identical plastid‐encoded β subunits (18–20 kDa) closely related to the β‐phycoerythrin of the red algal phycobilisome, combined with two similar but non‐identical “new α” subunits (Wilk et al., 1999). The new α_L_ and α_S_ subunits (for long and short, respectively; also known as α_1_ and α_2_) are small (8–10 kDa), nuclear‐encoded and completely unrelated to the phycobilisome α subunits (Wilk et al., 1999; Doust et al., 2004). The holoprotein is organized as a quasi‐symmetrical “dimer” of two αβ protomers, where the protomer is often referred to as a “monomer” by convention. The α_L_ subunit possesses a longer C‐terminal tail than the α_S_ subunit, a tail that partially covers the central β50/61 chromophore from the neighboring β subunit (*Note*: chromophores are labeled α or β indicating the subunit followed by the residue number of the cysteine to which they are covalently linked). In PE545, each α subunit binds a single 15,16‐dihydrobiliverdin (DBV) chromophore while the β subunits each bind three phycoerythrobilin (PEB) chromophores via thioether linkages. The structure of phycocyanin 645 (PC645) from *Chroomonas* sp. was later shown to have a very similar protein fold, in spite of the fact that the α subunits bind a different chromophore, mesobiliverdin (MBV), while the β subunits bind two types of bilin: one DBV (β50/61) and two phycocyanobilins (PCB; β82 and β158) (Harrop et al., 2014).

Cryptophyte algae synthesize a greater number of bilin chromophores than either the red algae or the cyanobacteria, resulting in light harvesting complexes exhibiting a wide variety of absorption maxima, spanning the spectral range between the red and blue absorption peaks of chlorophyll (Glazer & Wedemayer, 1995). To date, any one species appears to synthesize only one spectroscopically distinguishable type of phycobiliprotein. The phycobiliproteins have historically been named as either phycoerythrin (PE) or phycocyanin (PC) due to spectroscopic differences, despite the fact that the β subunits are only derived from red algal phycoerythrins, not phycocyanins (Apt et al., 1995). This naming convention does however correspond to either a phycoerythrobilin or a phycocyanobilin bound at Cys82 of the β subunit (Wedemayer et al., 1996), followed by the absorption maximum (Table 1). Cryptophytes are separated into five major clades on the basis of nuclear SSU rRNA sequences, which are partly reflected in their phycobiliprotein type (Hoef‐Emden, 2008). Members of clades 2, 4, and 5 all have PE545, Clade 3 has PE566, but in Clade 1 (*Chroomonas* and *Hemiselmis*) there are a variety of PCs as well as one PE555 (Table 1).

**TABLE 1:** Cryptophyte phycobiliproteins characterized by crystallography.

Name (culture collection number)	PBP	Pigment at position				Quaternary structure	Resolution	PDB accession code
		α19	β50/61	β82	β158			
*Rhodomonas* sp. (CS24)	PE545	DBV	PEB	PEB	PEB	closed	0.97 Å	1XG0
*Cryptomonas pyrenoidifera* (CCAP979/61)	PE566	bilin 612	bilin 584	PEB	bilin 584	closed	2.0 Å	7T8S
“*Chroomonas*” sp. (CCMP270)	PC645	MBV	DBV	PCB	PCB	closed	1.35 Å	4LMS
*Chroomonas gentoftensis* (CCAC1627)	PC630	MBV	DBV	PCB	PCB	closed	1.8 Å	7T7U
*Hemiselmis andersenii* (CCMP644)	PE555	PEB	DBV	PEB	PEB	open	1.8 Å	4LMX
*Hemiselmis virescens* (CCAC1635)	PC612	PCB	DBV	PCB	PCB	open	1.8 Å	4LM6
*Hemiselmis pacifica* (CCMP706)	PC577	PCB	DBV	PCB	PCB	open	1.0 Å	7T89

The discovery of long‐lived quantum coherence at room temperature in both *Rhodomonas* PE545 and *Chroomonas* PC645 (Wong et al., 2012; Turner et al., 2012; Collini et al., 2010) raised the question of whether coherent quantum processes play an important role in biology and stimulated renewed interest in the structure and evolution of these novel phycobiliproteins. Crystal structures from two *Hemiselmis* species (PC612 from *Hemiselmis virescens* and PE555 from *Hemiselmis andersenii*) revealed that they have a radically different quaternary structure caused by the insertion of a single aspartic acid residue into the α subunit sequence, just preceding the cysteine residue that is covalently attached to the bilin (Harrop et al., 2014). This single residue insertion resulted in a 73° rotation of the αβ protomers with respect to each other, producing a central cavity and separating the two central chromophores so they are no longer in van der Waals contact. This “open” form quaternary structure is correlated with the loss of the electronic coherence observed for the conventional “closed” form quaternary structure, where the latter structure is more compact and there is strong electronic coupling between the two central chromophores (Harrop et al., 2014; Arpin et al., 2015). However, the overall fold of the αβ protomers from all the crystal structures is very similar (Harrop et al., 2014).

Gene location and diversity creates another striking feature of the cryptophyte phycobiliproteins. Nearly all cryptophytes have a single, plastid‐encoded gene for the highly conserved β subunit (Douglas, 1992). In contrast, the α subunits are encoded by nuclear‐located multigene families (Curtis et al., 2012; Gould et al., 2007; Broughton et al., 2006). The only complete cryptophyte nuclear genome sequence, that of *Guillardia theta*, contains 20 α subunit genes (Curtis et al., 2012). Proteomic analysis shows that all 20 α subunit genes are expressed as proteins when *G. theta* is grown under white light of varying intensities (Kieselbach et al., 2018). The functional consequences of the large α subunit gene families are still unclear.

To better understand the evolution, genetic and spectral diversity among cryptophyte PBPs, we determined the crystal structures of three additional PBPs from different cryptophyte species covering the whole spectrum of phycobiliprotein types (Hoef‐Emden, 2008). These are: PC630 from *Chroomonas gentoftensis*, PC577 from *Hemiselmis pacifica*, and PE566 from *Cryptomonas pyrenoidifera*. In each case, the PBP can be paired with a previously determined structure with different spectral properties: PC645 from “*Chroomonas*” sp. (CCMP270), PC612 from *Hemiselmis virescens* (CCAC1635), and PE545 from *Rhodomonas* sp (CS24), respectively (Table 1).

In the case of the two PEs, we found that the overall structures of PE545 and PE566 are nearly identical, despite the fact that they bind chemically distinct chromophores. In particular, the spectral differences are due to a change in conjugation in the propionate side chain attached to pyrrole ring C which converts DBV to bilin 612, and PEB to bilin 584 in PE566. The remaining pairs, PC630‐PC645 and PC577‐PC612, have chemically identical chromophores within each pair, but different spectral properties (Arpin et al., 2015). The changes in spectral properties appear to be due to relative rotations of individual pyrrole rings within the chromophores and changes to the local environment around each chromophore. For each pair of proteins, these chromophore structural changes are due to sequence differences between the α subunits.

By examining all available α subunit sequences, we find that: *Hemiselmis* species contain α subunit genes for both open and closed form PBPs. Proteomic analysis shows that both forms are expressed in *H. virescens*. Finally, we find that each of the PE‐containing species has one α subunit sequence possessing a tandem pair of α‐like domains, with each domain containing all the characteristic features of a mature α subunit. These are likely to form a still undiscovered type of cryptophyte PBP which we have modeled using AlphaFold2 (Jumper et al., 2021).

## 2 RESULTS

### 2.1 General features of phycobiliprotein sequences

**FIGURE 1:**
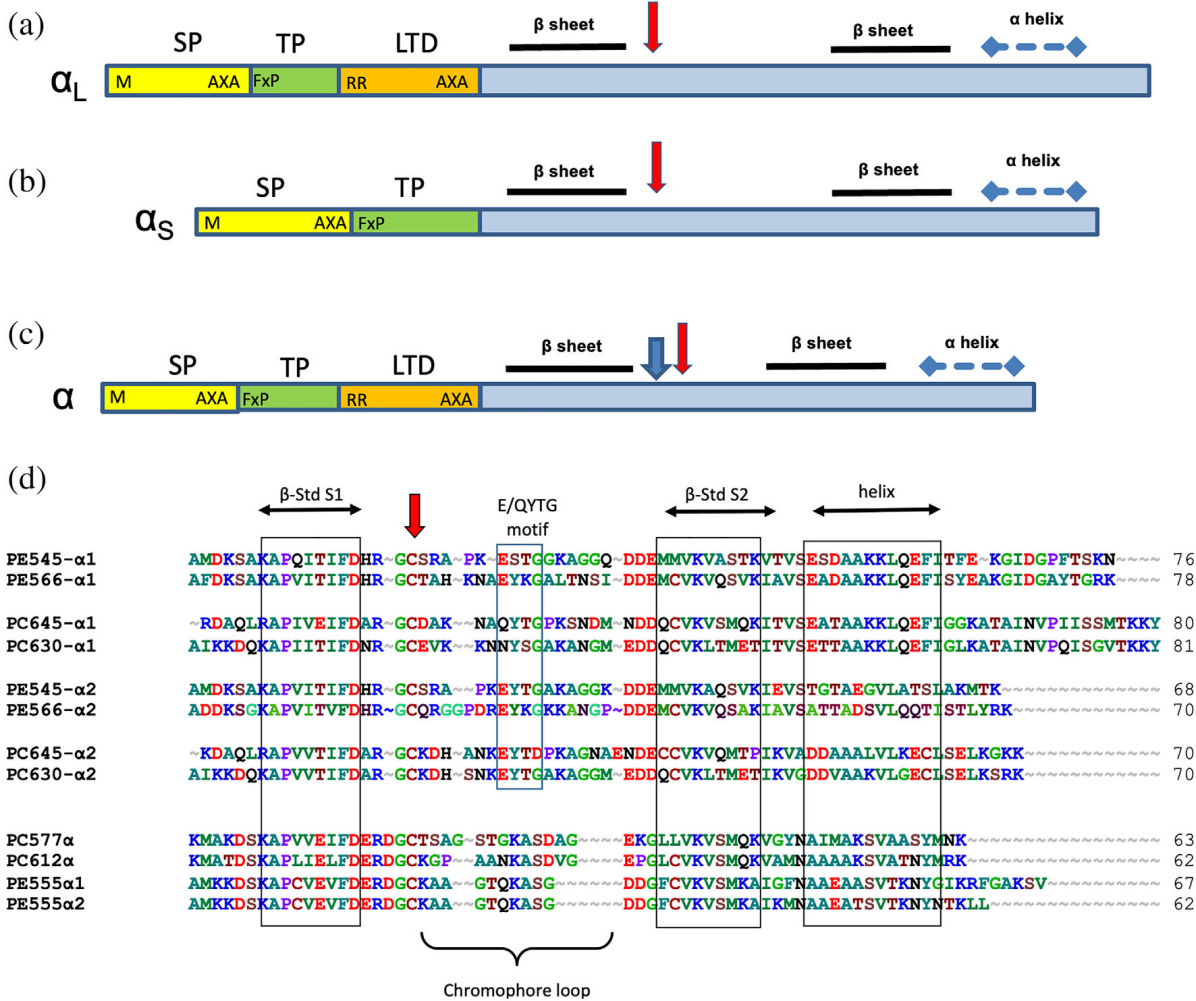
Structure of α subunit precursor proteins and alignment of mature α subunit sequences from x‐ray structures. (a–c) Precursors are synthesized on cytoplasmic ribosomes and directed across the endoplasmic reticulum by a typical ER signal peptide (SP) and then across three membranes into the plastid stroma where the transit peptide (TP) is removed. The lumenal targeting domain (LTD) with twin Arg motif (RR) directs the assembled and chromophorylated holoprotein into the thylakoid lumen where it is cleaved by the thylakoidal processing protease, leaving the mature protein (blue). (a) Typical α_L_ and (b) α_S_ sequences from *Rhodomonas* sp. CS24 PE545; (c) *Hemiselmis* α sequence with Asp insertion (blue arrowhead). Red arrow: bilin binding site. The variable chromophore loop region is between the bilin‐binding site and the second sheet region. (d) Alignment of mature α subunit sequences from x‐ray structures. Boxes indicate conserved blocks corresponding to secondary structure elements or specific sequence motifs. Amino acids are colored by side chain properties. Red arrow: Cys19 that attaches the bilin chromophore. PE545, *Rhodomonas* sp. CS24; PE566, *Cryptomonas pyrenoidifera* CCAP 979/61; PC645, “*Chroomonas*” sp. CCMP270; PC630, *Chroomonas gentoftensis* CCAC1627; PC577, *H. pacifica* CCMP706; PC612, *H. virescens* CCAC1635; PE555, *H. andersenii* CCMP644.

Full‐length α subunit gene sequences were obtained by PCR from nine species spanning all five of the major clades of cryptophyte algae (see Section 4). These sequences aided in the interpretation of crystallographic electron density maps and the subsequent structure determination and refinement. In general, several α_L_ and α_S_ sequences were obtained from each species. The exception was those in the genus *Hemiselmis* where most α subunit sequences have approximately the same length. As shown by Broughton et al. (2006) for *Rhodomonas* PE545 (Figure 1), all precursor α subunit proteins start with a typical ER signal peptide (SP), followed by a short plastid transit peptide (TP) similar to those found in other algae with secondary plastids (Huesgen et al., 2013). This is followed by an ER signal‐like lumenal targeting domain (LTD), ending in the sequence AxA, which marks the cleavage site for generating the mature α subunit. However, the α_S_ precursors of PE545 (Figure 1b) and PE566 are missing the LTD. Since the plastid‐encoded β subunits do not have an LTD either, the LTD of the α_L_ subunit alone must direct the completely assembled, folded and chromophorylated (α_L_β).(α_S_β) complex across the thylakoid membrane into the lumen (Gould et al., 2007; Broughton et al., 2006).

Comparison of the mature α subunit protein sequences, including those from the three newly solved structures (Figure 1d), shows that they all share conserved domains corresponding to the structural features found in previous investigations (Wilk et al., 1999; Doust et al., 2004; Harrop et al., 2014). All α subunit proteins form a β‐hairpin lying in a shallow groove along the surface of the β subunit. Between the two antiparallel β strands are the bilin‐binding motif (FDxRGC or FDxRDGC) and a variable surface loop region, called the chromophore loop, which covers the α subunit chromophore, shielding it from the aqueous environment. The C‐terminal α helices are involved in interactions between the two protomers and with the chromophore attached to the α subunit (Wilk et al., 1999; Doust et al., 2004; Harrop et al., 2014). The *Hemiselmis* sequences are shorter and have a single aspartic acid insertion two residues before the covalent chromophore attachment cysteine (Figure 1d). This single residue Asp insertion alone is responsible for the open form quaternary structure of the *Hemiselmis* (αβ)_2_ complex (Harrop et al., 2014).

### 2.2 Crystal structure of the closed form PC630 from *Chroomonas gentoftensis* CCAC1627 and comparison to its orthologue PC645 from “*Chroomonas*” sp. CCMP270

**FIGURE 2:**
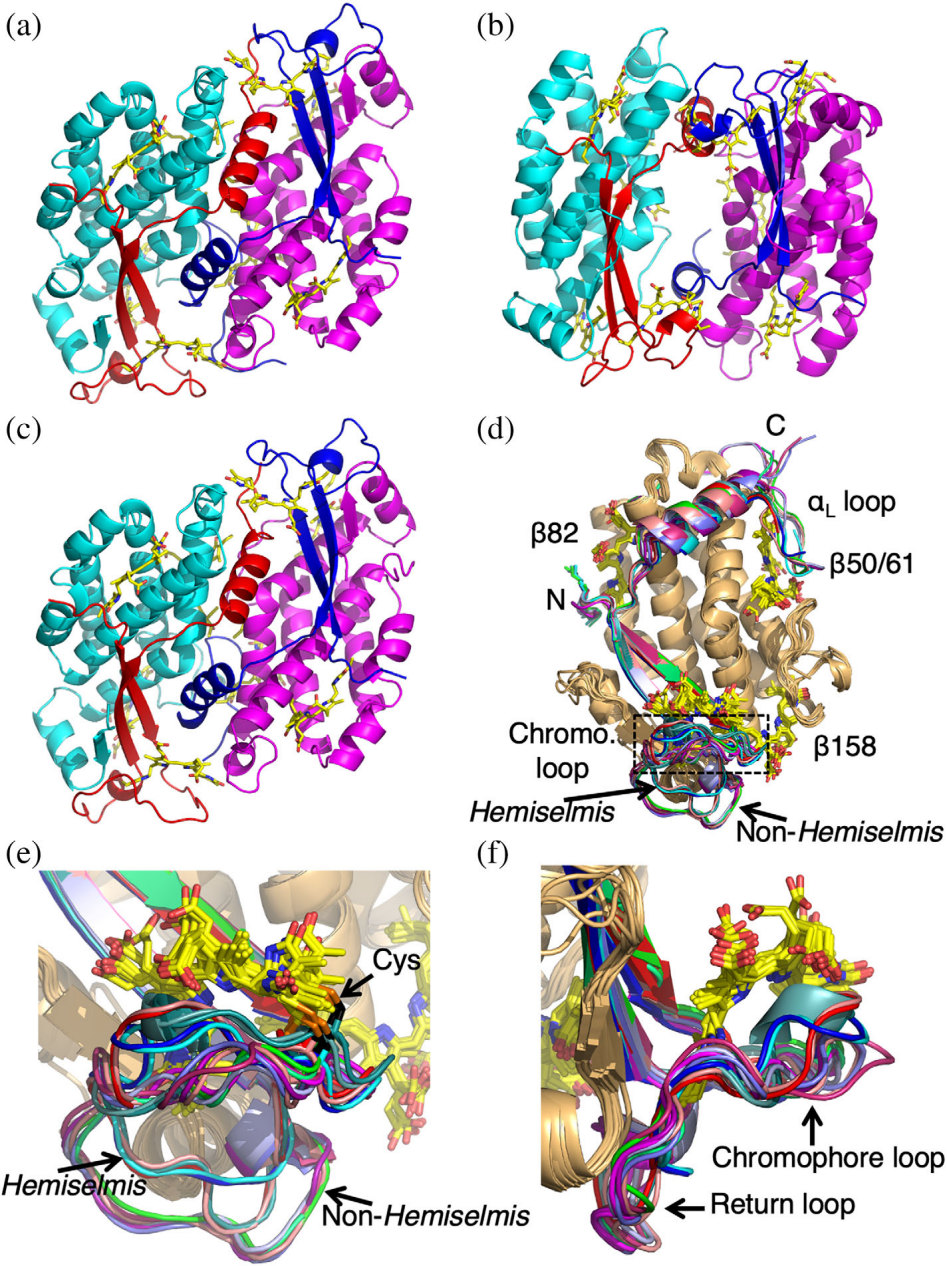
Crystal structures of cryptophyte light harvesting proteins. (a) Structure of the closed form PC630. (b) Structure of the open form PC577. (c**)** Structure of the closed form PE566. β subunits are colored magenta and cyan, while α subunits are colored blue and red. In closed form structures, α_L_ is colored blue while α_S_ is red. (d) An overlay of all unique αβ protomer structures determined to date. All β subunits are wheat. Chromophores are shown as stick models. (e) Expanded view of the overlay focusing on the α chromophore loop. The outward chromophore loop (middle, corresponding to the boxed area in panel (d)) from the cysteine (shown as sticks with C_β_ black and S_γ_ orange) to which the chromophore is attached follows many different paths. The return loop (bottom) shows three distinct paths: two for *Hemiselmis* and one non‐*Hemiselmis* structures. (f) The same overlay as per (e) but rotated by 90°. The outbound chromophore loop cradles the α chromophore while the return loop hugs the underlying β subunit.

The crystal structure of PC630 from *Chroomonas gentoftensis CCAC1627* was determined at 1.8 Å resolution (Table S1). The structure shows a closed form cryptophyte PBP (Figure 2a). This protein is very similar to the previously examined PC645 from “*Chroomonas*” sp. CCMP270 (RMSD 0.324 Å over 2732 atoms). The two complexes share the same chromophores (Table 1) and the β subunit sequences are nearly identical (168/177 residues). The α subunits are approximately 65% identical to their respective orthologues (α_L_ 52/81 residues identical; α_S_ 46/71 residues identical).

While the absorption spectra of the two proteins share main absorption peaks at 630 nm and 645 nm, the relative peak heights differ, with the 630 nm absorption peak larger in PC630 and the 645 nm peak larger in PC645, which led to the proteins being given different names (see fig. 1D in Arpin et al., 2015).

**FIGURE 3:**
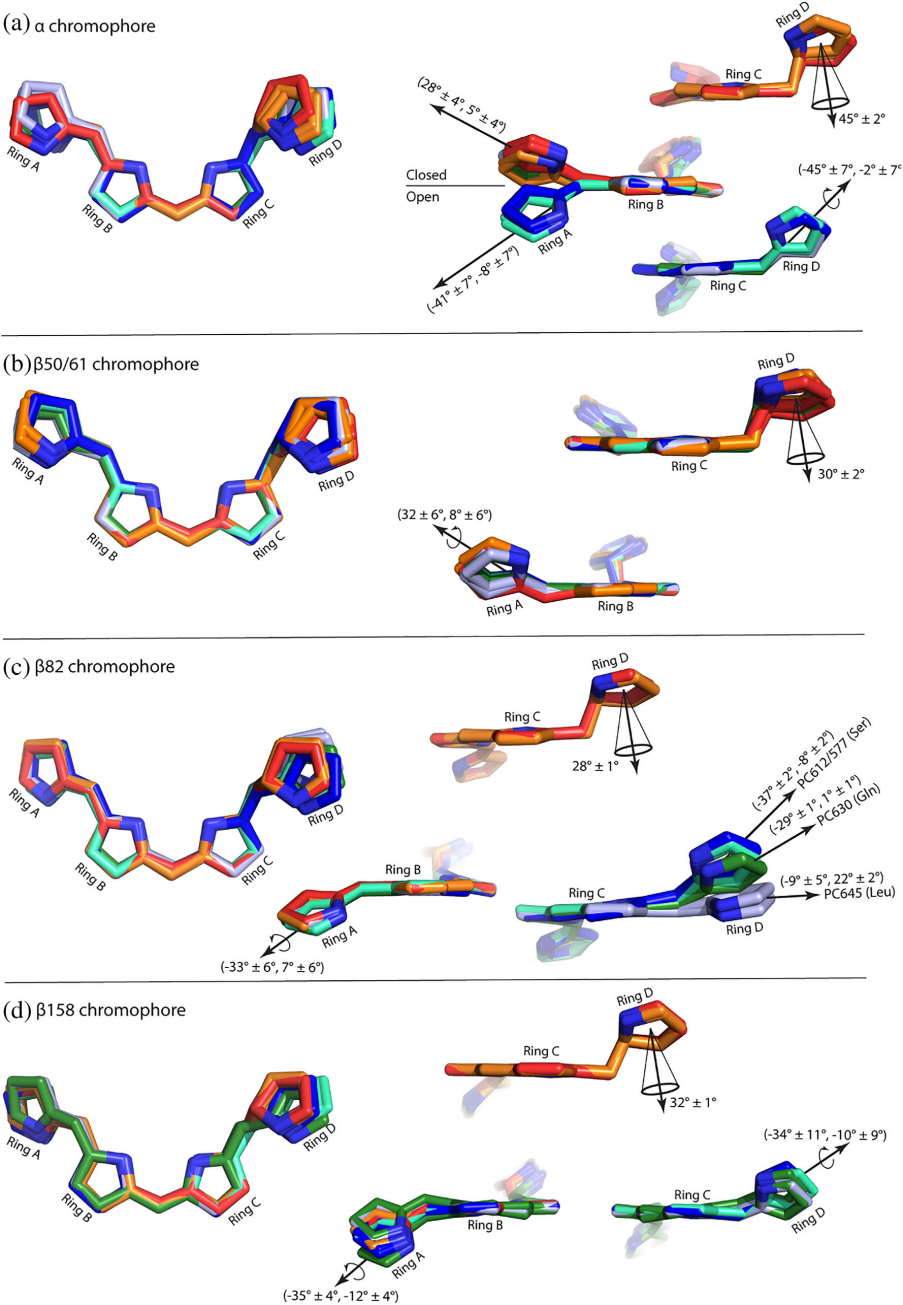
Chromophore geometry. An overlay of chromophores using the central pyrrole B and C rings for superposition. Chromophores have been stripped of any external chemical groups for visual simplicity. Chromophores are grouped by location in the structure, with (a) α chromophore, (b) bilin β50/61, (c) bilin β82, and (d) bilin β158. Only gross patterns are shown; some chromophores have finer hidden patterns that are explored in Figures S2–S6. The left‐hand side shows the overview overlay while the right‐hand side panels show details of pyrrole ring A and D rotations with reference to the planar central rings B and C. Conjugated and non‐conjugated chromophores are grouped for ring D as the analysis is different. Angle measures are either given as dihedral angle pairs (*θ*_inner_, *θ*_outer_) for conjugated ring pairs (see Section 4, with standard deviation taken over the set of all angles for each group) or as a single angle, *φ*, between two ring planes for non‐conjugated ring pairs (see Section 4, with standard deviation taken over the set of all angles for each group). In panel (c), right hand side, clusters are labeled based on the amino acid at residue α5/6 in the α subunit that interacts with the β82 chromophore. PC630 in dark green, PC577 in cyan, PC645 in light blue, PC612 in dark blue, PE545 in red and PE566 in orange. Standard deviation is over all members of each cluster.

**FIGURE 4:**
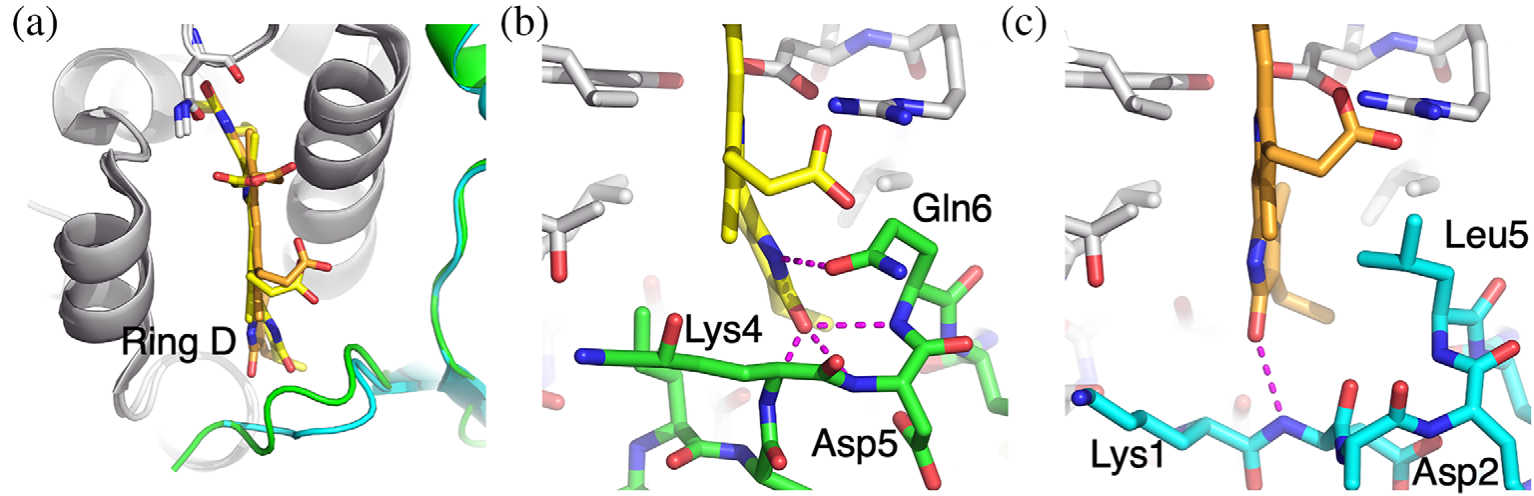
Sequence differences between the N‐termini of PC630 and PC645 α subunits rotate pyrrole ring D in the β82 PCB chromophore. Panel (a) shows an overlay of the β82 chromophore site where the PC630 chromophore is shown in yellow CPK while the PC645 chromophore is shown in orange CPK colors. PC630 α subunit is shown in green while the PC645 α subunit is cyan. (b) Multiple hydrogen bonds between PC630 α subunit (green CPK) and β82 pyrrole ring D stabilize the chromophore conformation. (c) PC645 α subunit forms one backbone hydrogen bond with pyrrole ring D, however, the side chain of Leu5 from the α subunit makes van der Waals contact, maintaining the chromophore conformation. The figure shows models for α_S_, however, the same interactions are seen in α_L_.

Although the chemical structures of all the chromophores are identical between PC645 and PC630 (Table 1), a careful examination of structural details showed that the key difference between them was a twist along the bond between pyrrole ring D and ring C in the PCB attached to β82. This can be seen when the chromophores are aligned via their central pyrrole rings B and C (Figure 3c), where β82 chromophores from PC645 and PC630 form distinct clusters. Twists are quantified by the two dihedral angles for the bonds linking adjacent pyrrole rings (similar to Ramachandran angles for peptide bonds, see Section 4; Figure S1). PC645 differs from PC630 by a large positive twist, as shown by differences in the dihedral angles (+20 ± 6°, +21 ± 3°) across the bridging bonds (Figures 3c and 4a and S4d, Table S4). Comparing all crystal structures of cryptophyte PBP αβ protomers shows that PC645 is the outlier with respect to the twist of pyrrole ring D in the chromophore attached to β82 (Figures 3c and S4d, Table S4).

The origin of this chromophore structural difference lies in the unique sequences of the N‐termini of the PC645 α subunits. In PC630, Gln6 in both α_L_ and α_S_ makes a side chain hydrogen bond to the nitrogen atom in pyrrole ring D of the β82 chromophore (Figure 4b). Additionally, the three backbone amide nitrogen atoms of residues Lys4, Asp5, and Gln6 make hydrogen bounds to the carbonyl oxygen on pyrrole ring D in both α_L_ and α_S_ (Figure 4b). In contrast, Gln6 of PC630 is replaced by Leu5 in each α subunit of PC645 (Figure 1d). Leu5 sterically prevents pyrrole ring D of chromophore β82 adopting the same orientation as all other cryptophyte structures (Figure 4c). In PC645, the only other interaction between the β82 chromophore and the α subunit is a single backbone hydrogen bond between the amide nitrogen of Asp2 and the carbonyl oxygen of pyrrole ring D (Figure 4c). Thus, the sequence of the α subunit N‐terminal segment dictates the twist of pyrrole ring D of the β82 phycocyanobilin (PCB) chromophore and hence the spectral difference between PC630 and PC645.

We note that the N‐terminal residues of the α_L_ and α_S_ subunits are near identical to each other within each organism but are markedly different between PC630 and PC645 (Figure 1d) even though the two genes coding for α_L_ and α_S_ subunits probably separated prior to speciation. It appears that the organisms have co‐evolved the N‐terminal regions of these closed form α subunits to maintain the conformation of the β82 chromophore.

Other differences between chromophore structures of PC630 and PC645 are subtle (Figures S2–S5). The α19 chromophore on PC630 α_L_ shows a very slight bowing between pyrrole rings B and C, which is not present in any of the other α19 chromophores in PC630 or PC645. This is likely to be due to sequence differences in the chromophore loop of PC630 α_L_ (Figure 1d) that result in the disruption of a conserved salt bridge that stabilizes the conformation of the α chromophore (see Section 2.8). In contrast, the α19 chromophores attached to α_S_ have coplanar pyrrole rings B and C in both PC645 and PC630, where the rings are supported by a salt bridge between His22 and Glu26. A similar supporting salt bridge structure is seen supporting the α19 chromophores in PE545 and PE566 for both α_L_ and α_S_ (see below).

Another minor feature is a chirality difference between the Cys50‐β50/61 thioether linkage in the α_L_β protomer versus the α_S_β protomer in both PC630 and PC645 (Figure S7). For both proteins, the thioether linkage in the α_L_β protomer is right‐handed (Figure S7a), whereas that in the α_S_β protomer is left‐handed (Figure S7b). This creates a difference in the orientation of pyrrole ring A in the β50/61 (Figure S3b), where PC645 chain B (PDB file 4LMS) and PC630 chain B correspond to the chromophore in the α_L_β protomer, with PC645 chain D and PC630 chain D on the α_S_β protomer. This structural asymmetry is due to differences in the chromophore environment, where in the α_L_β protomer, the β50/61 chromophore interacts with both the α_L_ C‐terminal loop and the β subunit GH loop, whereas in the α_S_β protomer the chromophore only interacts with the GH loop from the α_L_β protomer. This difference in thioether chirality and orientation of pyrrole ring A in the β50/61 chromophore is observed for both PC645 and PC630. It further emphasizes the effects of a multitude of small structural differences resulting from minor changes in amino acid sequences.

### 2.3 Crystal structure of the open form cryptophyte phycocyanin PC577 from *Hemiselmis pacifica* and comparison to orthologue PC612

The crystal structure of PC577 from *H. pacifica* CCMP706 has been determined at 1.0 Å resolution (Table S1). The structure shows a two‐fold symmetric (αβ)_2_ complex that adopts the open form quaternary structure (Figure 2b) which is closest to that of PC612 from *H. virescens* (RMSD 0.531 Å over 2909 atoms; cf., PE555 from *H. andersenii*—RMSD 0.670 Å over 2657 atoms). The amino acid sequence of PC577 is also closest to that of PC612 (β subunit: 168/177 residues identical; α subunit: 37/63 residues identical). We note that an independent structure of PC577 has recently been reported albeit at lower resolution (1.80 Å) (Spangler et al., 2022). The two structures are essentially identical (RMSD 0.215 Å over 334 C_α_ atoms).

The atomic resolution electron density map shows unequivocally that PC577 has the same chromophores as PC612 (Table 1). This confirms the identification of PCB on α20 and β82 and resolves the ambiguities in assigning β50/61 and β158 using mass spectrometry (Overkamp et al., 2014).

**FIGURE 5:**
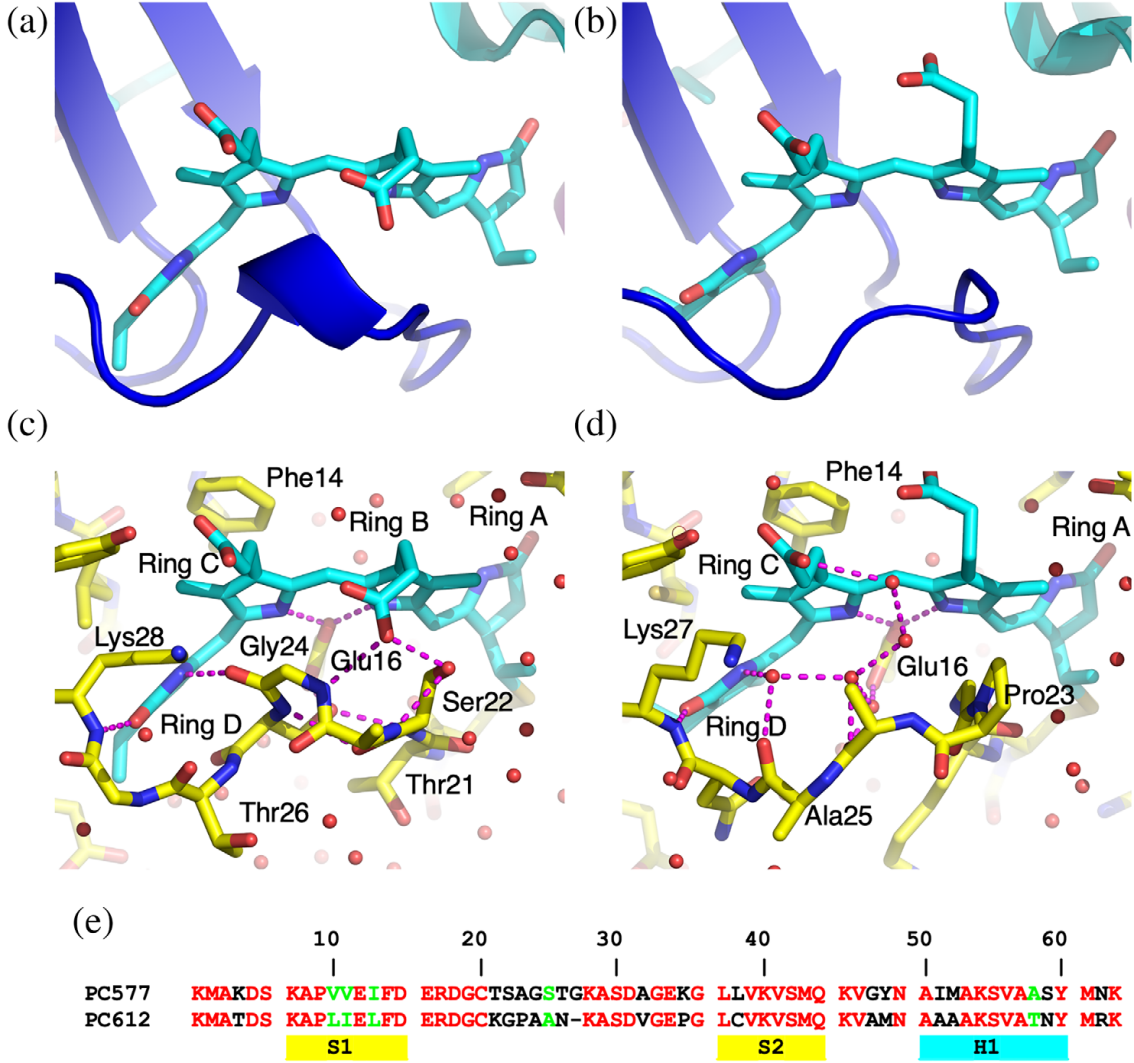
Sequence differences between PC577 and PC612 in the α subunit chromophore loop rotate pyrrole ring D in the α20 PCB chromophore. Panels (a, b) show cartoon representations of the α20 PCB chromophore site in PC577 and PC612, respectively. The chromophore loop of PC577 contains a one‐turn helix (a), which is not seen in PC612 (b). (c, d) Atomic models of the α20 chromophore site in PC577 and PC612, respectively. The view is identical to panels (a) and (b). In (c), the chromophore loop interacts with the α20 PCB where the backbone carbonyl of Gly24 (α subunit) makes a hydrogen bond with the nitrogen atom of pyrrole ring D, resulting in a rotation of this ring when compared to PC612 (panel (d)). Panel (d) shows the same view for PC612. Note, there is a gap between the α subunit chromophore loop and the chromophore surface, which is filled by ordered water molecules (red spheres). (e) Structure‐based sequence alignment of the α subunits of PC577 and PC612 (red—identical; and green—high similarity).

Although the absorbance spectra of PC577 and PC612 share the same features, the relative height of the two main peaks (577 nm and 612 nm) differs, again accounting for the distinct names (see fig. 1D in Arpin et al., 2015). Comparison of the chromophore structures shows subtle differences between PC577 and PC612 (Figure 5), which are more nuanced than the larger chromophore structural differences between PC630 and PC645 (Figure 4).

The structures of the β subunits from PC577 and PC612 are near identical, which is not surprising given the high level of sequence identity (168/177 residues). The chromophore structures are also near identical with only a slight twist between pyrrole ring C and ring D in the β158 PCB which is quantified by a difference in dihedral angles linking the rings: (−13 ± 6°, +12 ± 1°) (Figure 3d: Figures S5c,d and S8; Table S5). This pyrrole ring packs against the β ribbon in the α subunit, which is likely responsible for the slight alteration of conformation (Figure S8). The β ribbon in PC577 is closer to the β158 chromophore due to two substitutions in β strand S1: PC612 → PC577 Ile11 to Val11 and Leu13 to Ile13. Although pyrrole ring D of the β82 PCB chromophores interacts with the N‐termini of the α subunits (residues 2–6), the structures and interactions made by the α subunits of PC577 and PC612 are identical (Figure 1d; Figure S4d; Table S4), leaving the chromophore structure identical, in contrast to the differences seen between PC630 and PC645.

The largest structural difference between PC577 and PC612 is seen in the α subunit. The loop protecting the α20 PCB chromophore from the solvent is different in the two structures (Figure 5) and it is marked by a distinct sequence in each protein (residues 21–27 following Cys20, which is the covalent chromophore attachment site; Figure 5e). In PC577, this segment forms a single helical turn, making multiple hydrogen bonds to the α20 chromophore (Figures 5a and 5c). These hydrogen bonds couple the loop to several sites on the α20 chromophore constraining: the propionate group on pyrrole ring B, the nitrogen on pyrrole ring D, and the side chain of Glu16, which is the ligand for the two nitrogen atoms in the central pyrrole rings B and C (Figure 5c). Thus, the α20 PCB in PC577 is highly constrained by the protein. In contrast, in PC612, the equivalent segment of the chromophore loop separates from the chromophore surface, creating a cavity filled by ordered water molecules (Figures 5b and 5d). It is conceivable that the straight jacketing of the α20 PCB in PC577 alters the absorption strength of the chromophore, given the change in decay routes.

**FIGURE 6:**
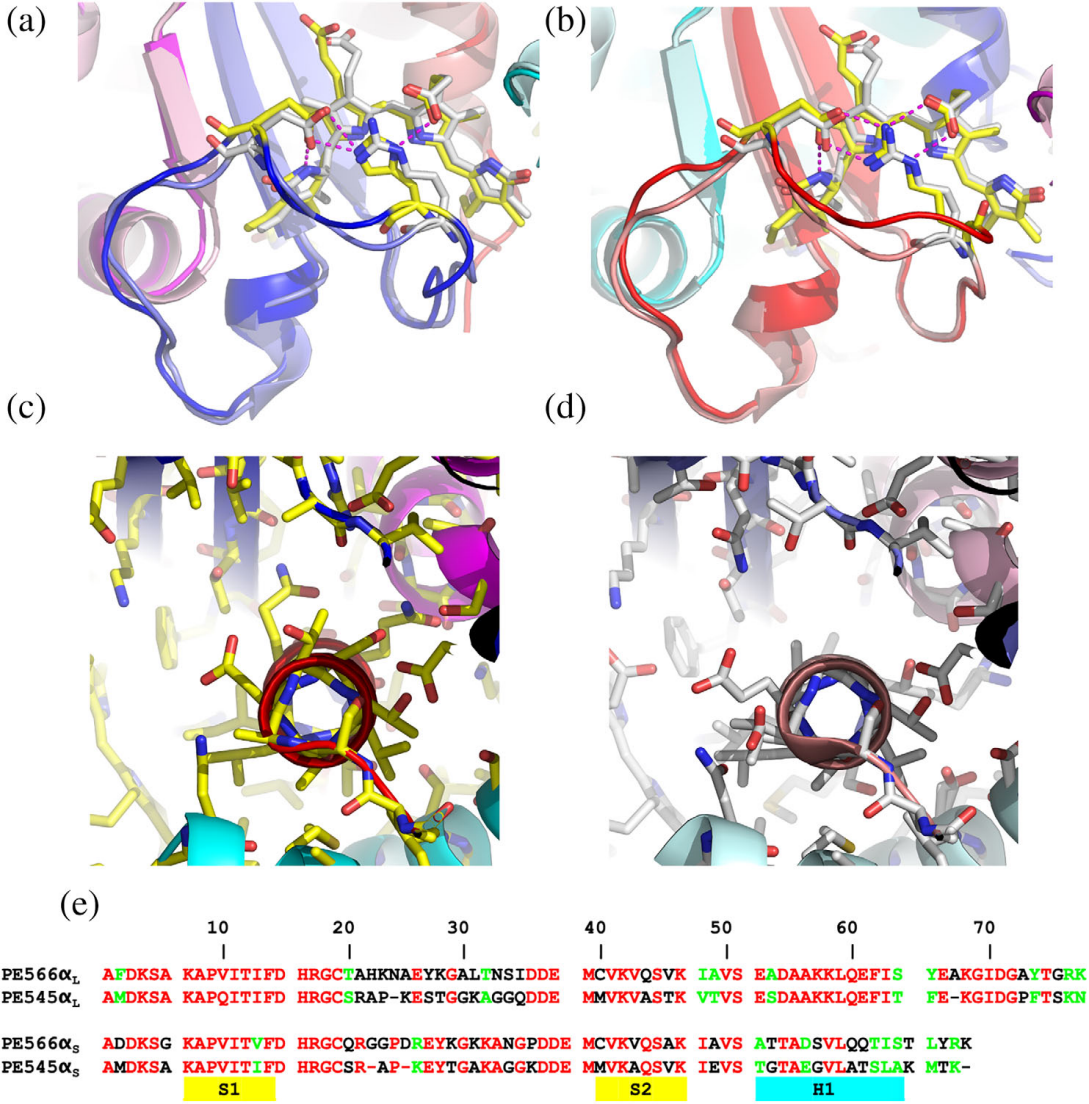
Comparison between PE566 and PE545. (a,b) Overlay the α subunit chromophore sites for α_L_ and α_S_, respectively. Although the chromophore loop chains differ, a planar salt bridge lies across the face of the central pyrrole rings B and C, maintaining the α chromophore in the same conformation. Panel (a) compares α_L_ structures. Here in PE545, Arg21 and Glu25 form a salt bridge, while in PE566, His22 replaces Arg21, while maintaining the same planar salt bridge structure. Panel (b) compares α_S_ where the Arg‐Glu salt bridge is formed in both proteins (PE545: Arg21‐Glu25; PE566: Arg21‐Glu27). (c**)** The α_S_ helix of PE566, showing an unremarkable distribution of side chains. (d) In contrast, the α_S_ helix of PE545 shows one face with essentially no side chains (either Gly or Ala, only). (e**)** Structure‐based alignment of α_L_ and α_S_ sequences for PE566 and PE545. Sequences are broken to indicate secondary structure boundaries. In (a) and (b), ribbons are colored by chain as per Figure 2a–c, with PE566 in full colors and PE545 in pastel shades. Stick figures are colored with carbon gray for PE545 and carbon yellow for PE566.

In addition, the conformation of the α20 chromophore in PC577 differs from that of PC612 (Figure S2; Table S2). The backbone carbonyl group of Gly24 in PC577 forms a hydrogen bond to the nitrogen atom of pyrrole ring D in the α20 chromophore (Figure 5C). This results in a −10° rotation of the inner dihedral angle linking ring C to ring D in PC577 when compared to PC612 (Table S2, Figures 3a and S2). This is the largest deviation in chromophore structure between these two proteins.

Thus, the differences in amino acid sequence between the α subunits of PC577 and PC612 are likely to be responsible for the observed spectral differences. In this sense, the α subunits tune the absorption maxima of these two related proteins.

### 2.4 The crystal structure of phycoerythrin PE566 from *Cryptomonas pyrenoidifera* CCAP 979/61 at 2.0 Å and comparison to orthologue PE545

The crystal structure of PE566 has four copies of the closed form (α_L_β).(α_S_β) complex in the asymmetric unit (Figure 2c and Table S1). Each copy is nearly identical (RMSD <0.1 Å). The structure of PE566 is most like that of PE545 (RMSD 0.23 Å over C_α_ 266 atoms), with near identity between the β subunits (167/178 residues) and significant identity in the α subunits (sequence identity: α_L_—48/78 residues; and α_S_—39/70 residues; Figure 1d).

In contrast to the two previous examples, the spectral difference between PE566 and PE545 is primarily due to different chromophores. PE545 has DBV as the α subunit chromophore and PEB on the three β subunit chromophore sites (β50/61, β82, and β158). In contrast, the α19 chromophore of PE566 is bilin 618 and two sites on the β subunit, β50/61 and β158, have bilin 584 chromophores, while the β82 chromophore is PEB, as per PE545 (*Note*: the PE566 β subunit has an additional residue at the N‐terminus, hence the bilins are actually attached to Cys51, Cys62, Cys83, and Cys159, however, we keep the conventional bilin nomenclature to facilitate comparison between proteins). These chromophores correspond to those previously determined via ^1^H‐NMR and mass spectrometry (Wedemayer et al., 1991, 1992). We note that the presence of a double bond between the two central carbon atoms in the propionate side chains of pyrrole ring C in bilin 618 and bilin 584 is the only chemical feature that distinguishes these chromophores from DBV and PEB, respectively. These differences are responsible for the planarity of the propionate side chains in the electron density maps.

Comparing the structures of PE566 and PE545 shows only a few distinguishing features. Sequence differences in the β subunits tend to lie on the protein surface with minimal impact on the observed structures. One notable difference is in the loop between helices hG and hH which differs in the β subunit attached to α_L_ in the two structures. This loop interacts with pyrrole ring D in the β50/61 chromophore of its neighboring β subunit with the aliphatic portion of Lys151 making van der Waals contact with the face of pyrrole ring D. In contrast, this surface is solvent exposed in PE545.

The backbones of the two PE566 α subunits show very little difference when compared to PE545 (α_L_: RMSD 0.30 Å on 54 C_α_ atoms; α_S_: 0.39 Å on 57 C_α_ atoms). Despite having no sequence similarity and being one residue longer (Figure 1d), the structure of the chromophore loop (residues Thr19 to Ile34) in PE566 α_L_ is nearly identical to that of PE545 with PE566 His22 and Glu26 forming a salt bridge that supports the central pyrrole rings of the α19 chromophore (vs. Arg21 and Glu25 in PE545; Figure 6a) and the side chain of Glu26 making a hydrogen bond to the nitrogen in pyrrole ring D, as seen in PE545. The chromophore loop in PE566 α_S_ is two residues longer than in PE545, resulting in significant differences in the backbone, however, the salt bridge in PE566 between Arg21 and Glu27 supports the central pyrrole rings of the α19 chromophore in the same manner as Arg21 and Glu25 in PE545, and Glu27 in PE566 makes a side chain hydrogen bond to the nitrogen in pyrrole ring D of the α19 chromophore, as seen in PE545 (Figure 6b). We note that a similar salt bridge (Glu‐His) supporting the α chromophore is present in the α_s_ structures of PC645 and PC630 and it is a conserved feature of closed form α subunits (see Section 2.8).

Comparing the sequences of α_L_ between PE566 and PE545 (Figure 6e), the differences are largely concentrated in two regions: the chromophore loop (2/16 residues identical) and C‐terminal loop that interacts with the β50/61 chromophore of the adjacent β subunit (7/15 residues identical). In contrast, the differences between the α_S_ subunits are clustered in the C‐terminal region starting with the α helix (4/18 residues identical). The non‐conserved residues in the α helix lie on an exposed face (Figure 6c,d). In PE545, these residues are invariably small residues: Gly52, Gly56, Ala59 and Ala63, creating a water‐filled slot through the center of PE545 (Figure 6d), which is not as evident in other closed form cryptophyte PBPs (Wilk et al., 1999; Harrop et al., 2014).

### 2.5 Global comparison of all αβ protomer structures determined

Overlaying all the structures of the αβ protomer that have been determined to date (Figure 2d) reveals that the structures are nearly identical, in particular, the β subunit (Figure 2d β subunits wheat). Even the chromophore structures, where the chromophores are chemically diverse, show reasonable structural conservation apart from the changes noted in the preceding sections and the difference seen in pyrrole ring A of the α subunit chromophore that differentiates the open and closed form (Figure S2a,b) (Harrop et al., 2014). The major structural differences lie in the α subunits, particularly the loop that cradles the α chromophore (between the covalent attachment site for the α subunit chromophore and the second β‐strand in the α subunit) and the C‐terminal loop, which is particular to the closed form long α_L_ chain. The variation in the chromophore‐proximal portion of the α subunit chromophore loop shows wide backbone variation (Figure 2d boxed segment; Figures 2e,f) while the return loop to β strand S2 forms two tight clusters, one for *Hemiselmis* and the other for non‐*Hemiselmis* subunits (Figures 2e,f).

### 2.6 The α subunits are encoded by a large diverse gene family

The *G. theta* genome contains 20 different genes encoding PE545 α subunits, showing that in this species they make up a large gene family (Curtis et al., 2012; Gould et al., 2007). Furthermore, tryptic peptide sequencing showed that all 20 of these genes are not only transcribed but are significantly expressed at the protein level under several different light conditions (Kieselbach et al., 2018). This suggested that the small number of sequences identified by PCR (some of which were not identical to those in the crystallographic structures) might reflect preferential expression of a few members of a more diverse gene family, and the potential for formation of other types of (α_L_β).(α_S_β) complexes with different spectroscopic properties.

A search of the cryptophyte transcriptomes that became available through the Moore Foundation Eukaryotic Transcriptomes project (Keeling et al., 2014) yielded 9–23 unique transcripts per species (Table 2). In four cases (*H. virescens*, *H. andersenii*, *C. mesostigmatica*, and *P. sulcata*), the same species, although not the same strain, were used for our original PCR‐based sequencing. Homologs of most of the sequences determined from PCR were found in the transcriptomes, and in some but not all cases, they were among those most highly expressed.

**TABLE 2:** Number of α subunit genes from transcripts, RT‐PCR sequencing or genomics.

	PBP type	Alpha sequences from transcriptomes		PCR only
		Open	Closed	
CLADE 1 (open and closed)^a^				
*Hemiselmis andersenii* (CCMP644)	PE555	9	6	3 open
*Hemiselmis tepida* (CCMP443)	PE615^b^	9	6	
*Hemiselmis virescens* (PCC157/M1635)	PC612	6	3	3 open
*Hemiselmis rufescens* (PCC563)	PE555	15	5	
*Hemiselmis pacifica* (CCMP706)	PC577			3 open
*Hemiselmis cryptochromatica* (CCMP1181)	PE569^c^			3 closed
“*Chroomonas*” sp. (CCMP 270)	PC645			3
*Chroomonas mesostigmatica* (CCMP 1168) (CCMP269)	PC645		15	5
*Chroomonas gentoftensis* (CCAC1627)	PC630			5
*Chroomonas* sp. (CCAC1312)	PC630			4
PE CLADES (2–5)				
*Rhodomonas* sp. (CS24)	PE545			6
*Rhodomonas minuta* (CPCC344)	PE545			6
*Rhodomonas salina* (CCMP1319)	PE545		20	
*Guillardia theta* (CCMP2712)	PE545		20^d^	
*Hanusia phi*	PE545		13	
*Proteomonas sulcata* (CCMP705)	PE545		13	6
*Gemingera cryophila* (CCMP2564)	PE545		23	
*Cryptomonas pyrenoidifera* (CCAP979/61)	PE566			5
*Cryptomonas curvata* (CCAP979/52)	PE566		23	

^a^ All the sequences are closed except the *Helmiselmis* ones noted.

^b^ Lane and Archibald (2008).

^c^ Heidenreich and Richardson (2020) and Cunningham et al. (2019).

^d^ *G. theta* genome (Curtis et al., 2012; Kieselbach et al., 2018).

The most surprising finding was that in all the *Hemiselmis* strains examined (four species, seven isolates) there were transcripts encoding closed form α subunits characteristic of their *Chroomonas* relatives, rather than the open form found by PCR and represented in our crystal structures (this work; Harrop et al., 2014). They did not have the Asp insertion just prior to the bilin binding site, were generally longer than the open form α's, and resembled their counterparts from the *Chroomonas* spp. These closed forms were not minor components of the transcriptome but accounted for more than 40% of non‐redundant transcripts.

**FIGURE 7:**
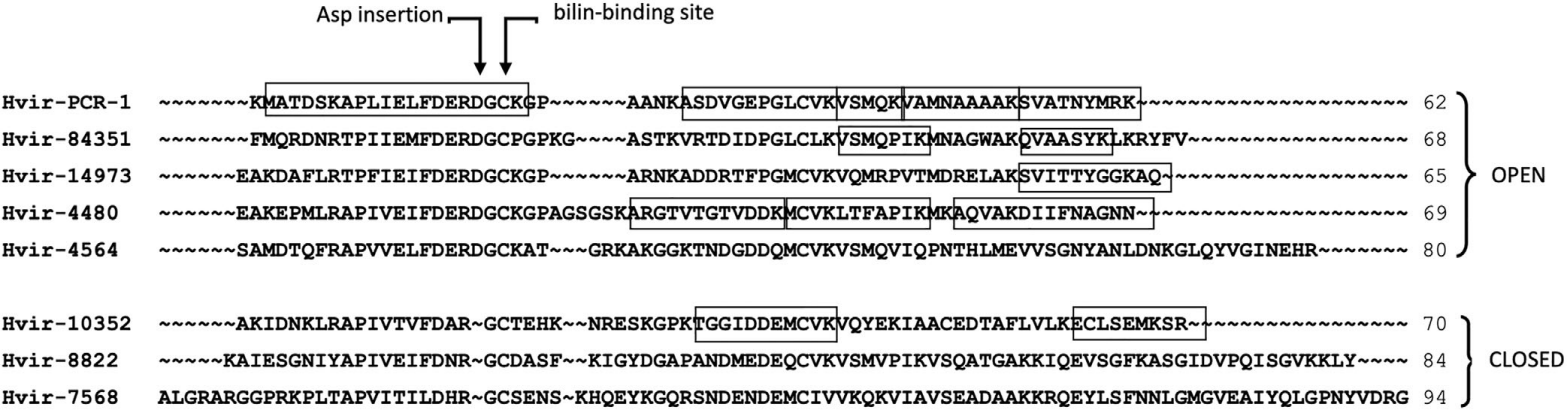
Tryptic peptides from *H. virescens* CCMP706 show that at least one closed form gene is expressed. Each peptide sequence found by LC–MS/MS is shown as a boxed section in its respective transcript sequence. Sequences are identified by their MMETSP1356 transcript number except for Hvir‐PCR‐1, the PCR‐derived sequence which is identical to the crystal structure sequence and to transcripts Hvir‐0052 and Hvir‐10022 (not shown). Note that the closed form sequence Hvir‐10352 is identified by two unique peptides. This sequence clusters with *Chroomonas*‐*Hemiselmis* α_S_ while the other two closed form sequences (Hvir‐8822 and Hvir‐7568) cluster with *Chroomonas*‐*Hemiselmis* α_L_ (see Figure 8). Dashes in the sequences are introduced for alignment.

As a first step to see if any of the three *H. virescens* transcripts predicted to form closed structures were expressed into protein, a soluble protein extract was fractionated by electrophoresis and the 8–10 kDa band containing the α subunits subjected to LC–MS/MS analysis. The predominant peptides found were those corresponding to the crystallographic structure, with almost complete coverage of the mature protein sequence (Figure 7). The other four proteins identified included three predicted to form the open configuration and one predicted to form the closed configuration. This typical α_S_ sequence was supported by two unique peptides. This suggests that one or more of the minor peaks discarded during protein purification for crystallography might represent (α_L_β).(α_S_β) complexes with closed form structures and different spectroscopic properties.

### 2.7 Sequence comparison and phylogeny

Over 200 translated protein sequences (from genomes, transcriptomes and manual sequencing) were aligned with MAFFT ver.7 (Kato & Standley, 2013) using the JTT200 substitution matrix (Figure S9). All sequences had the relatively conserved β‐sheet and chromophore‐binding motifs, but there was considerable variety in the length and composition of the N‐ and C‐terminal segments and in the chromophore binding loop. Many of the sequences included the entire presequence with ER domain, transit peptide, and LTD (Figure 1). Surprisingly, many of the α_2_'s do have LTDs, in contrast to what was found in earlier studies (Gould et al., 2007; Broughton et al., 2006).

Overall, the sequences fell into six to eight major groups (Figure S9). Most of the closed form *Hemiselmis* sequences grouped with either α_L_ or α_S_ homologs in *Chroomonas*. This included the three closed form sequences obtained by PCR from *Hemiselmis cryptochromatica*, a species that is almost colorless and only expresses a small amount of phycobiliprotein (Heidenreich & Richardson, 2020; Cunningham et al., 2019). The open form *Hemiselmis* sequences formed a completely separate group from all the closed form sequences although they have the same basic motifs; they were therefore omitted from Figure S9.

**FIGURE 8:**
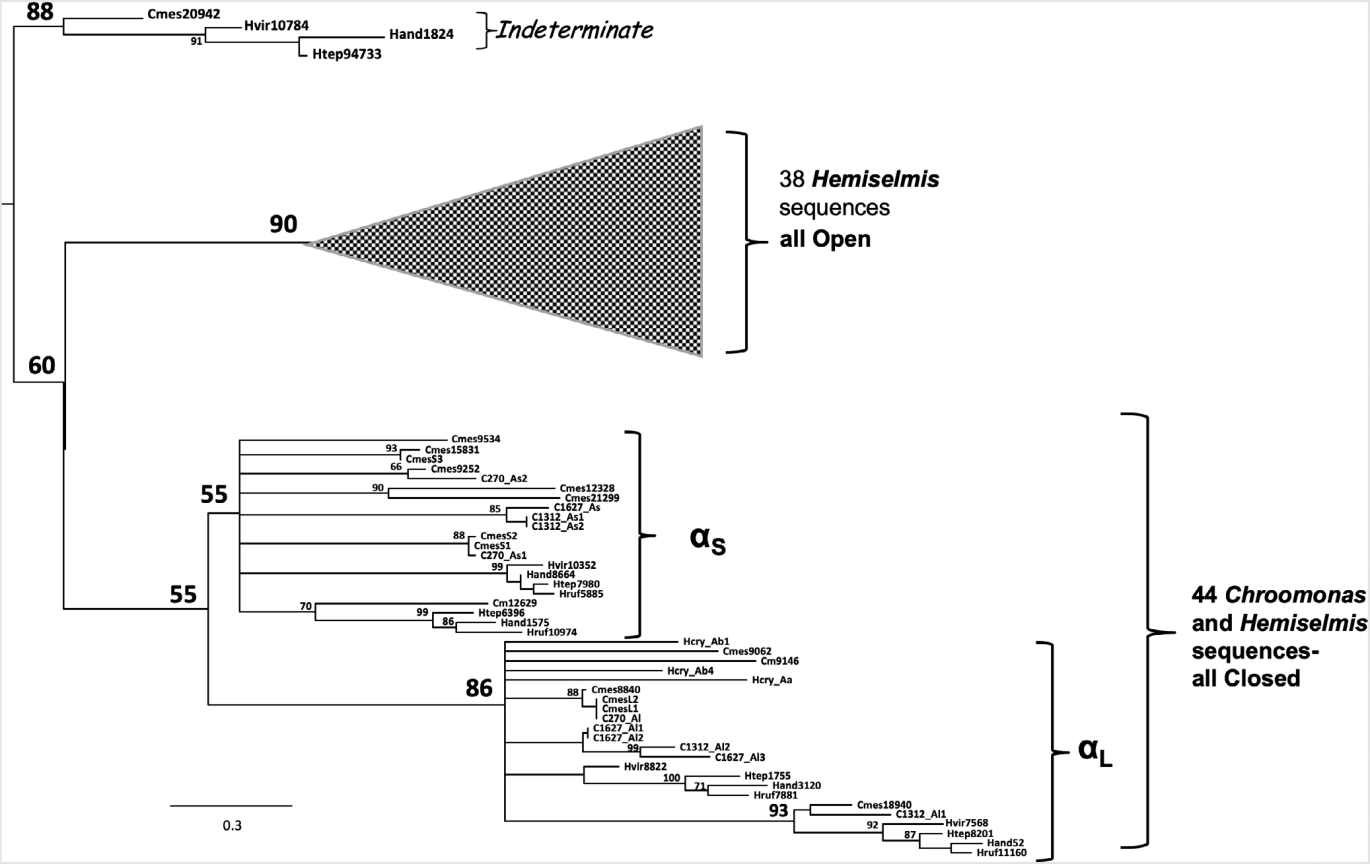
Phylogenetic tree of *Chroomonas* and *Hemiselmis* α‐subunit sequences shows open and closed forms are clearly distinguished. Open form *Hemiselmis* sequences form a single clade with a bootstrap support of 90. Closed form sequences break into α_L_ and α_S_ clades, where *Hemiselmis* sequences are grouped with those from *Chroomonas*. Four outlier sequences are classified as “indeterminate”. Cmes, *Chroomonas mesostigmatica*; C270, *Chroomonas* sp. CCMP270; C1312, *Chroomonas* sp. CCAC1312; C1627, C. *gentoftensis*; H. vir, *Hemiselmis virescens*; *H.and, H. andersenii; H. tep, H. tepida; H. ruf, H. rufescens; H. cry, H. cryptochromatica*. Scale bar: changes per 100 residues.

Phylogenetic analysis is difficult with families of small proteins (under 100 amino acids) because there are not enough phylogenetically informative positions to get strong statistical support. The best trees were obtained with RAxML (Stamatakis, 2014), but bootstrap percentages were low and no clear picture emerged after a number of attempts with various parameters. However, when the analysis was limited to the *Chroomonas*‐*Hemiselmis* clade (Clade 1), the open form sequences made a well‐supported branch separate from the closed form sequences (Figure 8) despite the fact that open forms have more variation in sequence than all of the closed forms together, particularly in the chromophore binding loop. Most of the *Hemiselmis* closed forms align with their *Chroomonas* homologs in two separate α_l_ and α_s_ branches. This suggests that the single residue Asp insertional mutation that gave rise to the open form happened early in the diversification of Clade 1. It is important to note that organismal trees based on nuclear and nuclemorph rRNA sequences show that *Hemiselmis* spp. form a well‐supported sub‐clade within Clade 1 but the genus *Chroomonas* is clearly polyphyletic (Hoef‐Emden, 2008). A thorough taxonomic analysis that included sequences obtained from hundred‐year old museum specimens of the type species *C. nordstedtii* showed that only strains containing PC630 are properly defined as *Chroomonas* (Hoef‐Emden, 2018); that is why “*Chroomonas*” with PC645 has quotation marks in our paper.

### 2.8 Conserved sequence motifs

The large sequence alignment (Figure S9) revealed several conserved motifs in the closed form α subunits. Previous studies identified a unifying CALM domain (cryptophyte α‐like motif), which includes the β‐hairpin secondary structure (Rathbone et al., 2021). Two additional conserved motifs can be seen in the sequence alignment. These are: EYxG between the two β strands in the chromophore loop; and KGIDG/TAINV/SGIDV within the C‐terminal loop of α_L_ (also known as α_1_) subunits beyond the α helix (Figure 2d).

The EYxG motif lies in the region of the chromophore loop adjacent to the α chromophore (Figure 2d, within the boxed region; Figure 2e,f). The hydroxyl group of the tyrosine residue forms the center of a hydrogen bonding network that connects the EYxG motif to the N‐terminus of helix Y of the β subunit forming an N‐cap (Figure S10). This motif is not present in open form α subunits. We note that in the case of *Rhodomonas* PE545 α_L_ (but not α_S_), the tyrosine is replaced by a serine and hence a water molecule forms the N‐cap of helix Y (Figure S10d).

The glutamic acid residue (sometimes glutamine) of the EYxG motif forms a salt bridge that lies across the face of the α chromophore (Figure 6a,b; Figure S11). Its partner is usually either an arginine or a histidine residue which is normally found two to three residues after the cysteine to which the chromophore is covalently bound (Figure S9). The glutamic acid also forms a hydrogen bond to the nitrogen atom of pyrrole ring D of the α chromophore (Figure S11). This structure is seen in all closed from structures to date with two variations. First, in PC630 α_L_ the glutamic acid is replaced by Asn25 while Lys23 forms a salt bridge to the propionate group on pyrrole ring B of the α chromophore (Figure S11c). This appears to be the cause of the distortion in the planarity of the central pyrrole rings of this chromophore (noted above). The second variation is PC645 α_L_ where the glutamic acid is replaced by Gln24 which makes a hydrogen bond to Asn22 and the nitrogen atom of pyrrole ring D (Figure S11d).

The KGIDG/TAINV/SGIDV motif is only observed in closed form α_L_ sequences. It forms the apex of the C‐terminal loop and lies over pyrrole ring A of the β50/61 chromophore from the β subunit in the α_s_β protomer. In the α_L_ PE group (Figure S9), the motif is KGIDG. The lysine residue forms hydrogen bonds with the carbonyl groups from both the glycine and aspartic acid (Figure S12a). This results in the side chain of the aspartic acid group pointing towards the β50/61 chromophore, where it forms a hydrogen bond to the nitrogen atom in pyrrole ring A (Figure S12c). In the α_L_ *Chromonas*‐*Hemiselmis* group (Figure S9), the motif is either TAINV or SGIDV. The initial threonine/serine can only make a hydrogen bond to the carbonyl group of the subsequent alanine/glycine (Figure S12b). As a result, the asparagine/aspartate side chain remains in the plane of the loop and it does not interact with the underlying chromophore (Figure S12d).

### 2.9 A conserved sequence with a tandem α subunit domain unites the PE clades

**FIGURE 9:**
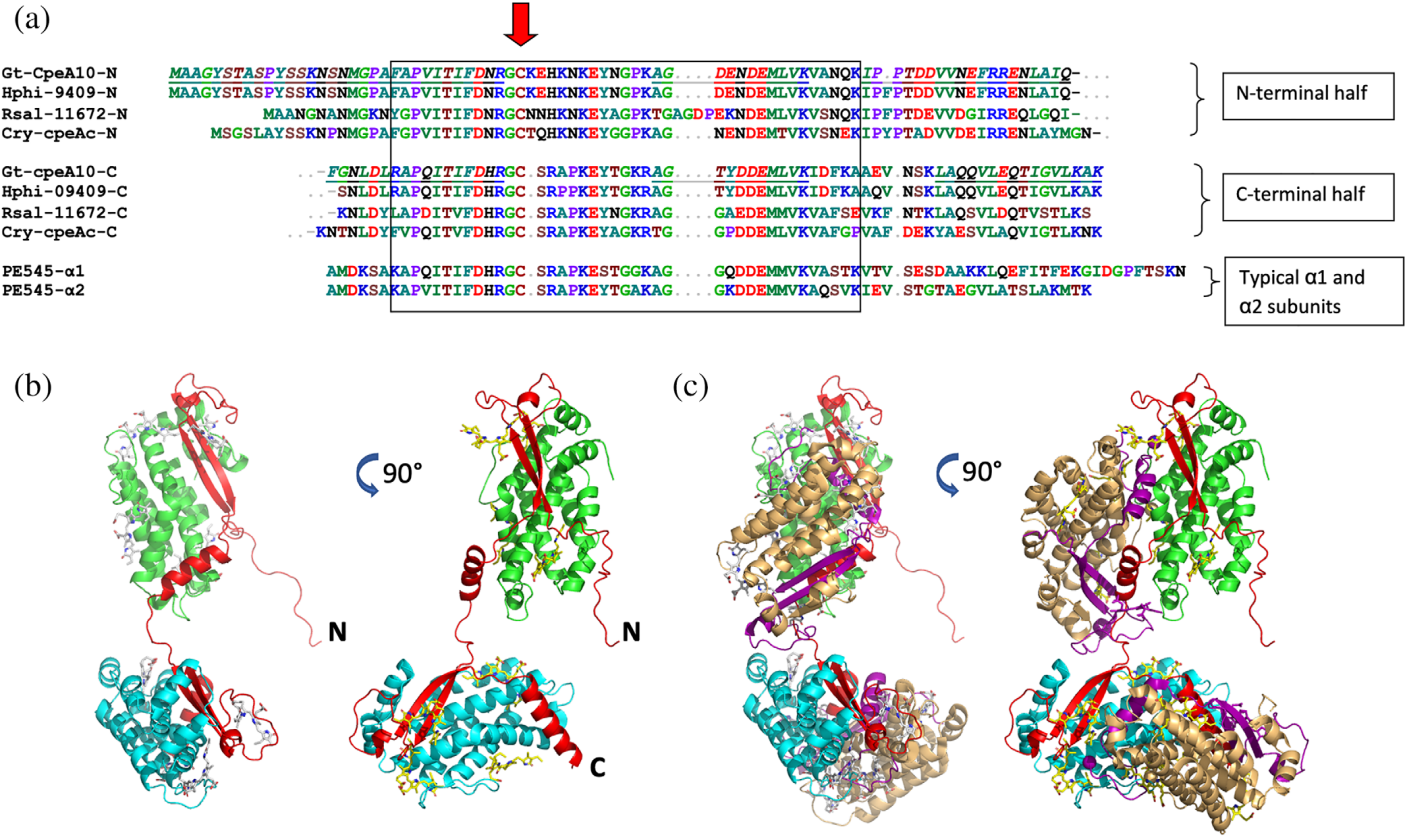
Internal duplication creating tandem α sequence conserved in four PE species from three clades. (a) The four sequences have been split into N‐terminal half (N) and C‐terminal half (C) to show the duplication (boxed). *G. theta* CCMP2712 CpeA10 peptide sequences identified via LC–MS/MS (Kieselbach et al., 2018) are underlined and italicized. (b) AlphaFold2 model of *G. theta* CpeA10 (red) bound to two β subunits (green and cyan). (c) Addition of two α_L_β protomers (α_L_ subunit purple, β subunit wheat) to create two complete complexes that are linked by CpeA10. Two views in (b) and (c) are related by a 90° rotation about the vertical axis in the page. The four tandem sequences are: Gt‐CpeA10, *G. theta* CCMP2712; Hphi, *Hanusia phi* CCMP325 MMETSP1048 transcript; Rsal, *Rhodomonas salina* CCMP1319 MMETSP1047 transcript; and Cry‐cpeAc PCR sequence from *Cryptomonas pyrenoidifera* CCAP 979/61. *Rhodomonas* sp. CS24 PE545 single α domain sequences are included for comparison. Red arrows, chromophore attachment cysteine site.

A single sequence with two chromophore‐binding α subunit motifs, each resembling a mature, closed form α_S_ subunit, was found by PCR in *C. pyrenoidifera*, matching the identical sequence (Cry_16635) found in the *C. curvata* transcriptome. Sequences with similar internal duplications of the mature α subunit were found in the transcriptomes of *R. salina* and *Hanusia phi*, and in the genome of *G. theta* (Figure 9a). The *G. theta* sequence was verified by peptide sequencing (Kieselbach et al., 2018). The fact that this tandem α sequence was found in *Cryptomonas*, which binds bilin 584 and bilin 618 rather than PEB, as well as in three other species with PE545, suggests that this tandem α subunit originated from a partial gene duplication in a common ancestor of these genera. No homologous sequences were found among the transcriptomes of *Hemiselmis* or *Chroomonas* species.

We have built a model for this new protein using the program AlphaFold2 (Jumper et al., 2021) which was initiated with two β subunits plus the tandem α subunit from *G. theta* (Figure 9b). The 25 default models calculated by Alphafold2 all show two αβ domains that are essentially identical to the αβ protomers observed in the crystal structures. The relative orientation of the two αβ domains in the complex varies between models, indicating that the sequence linking the two domains is flexible. The resultant model resembles the CaRSP scaffolding complexes observed in red algal phycobilisomes (Rathbone et al., 2021). All chromophore sites are maintained in the model even though AlphaFold2 does not include chromophores.

Given the hydrophobic surface that would be exposed at the chromophore sites, it is possible that this α_tandem_ββ complex binds two additional α_L_β protomers to mimic two mature cryptophyte α_L_β.α_S_β complexes, as shown in Figure 9c. We tested whether AlphaFold2 could assemble such a complex by initiating it with four β subunits, two α_L_ subunit sequences plus the tandem α subunit. All 25 default models created two non‐interacting complexes, one containing two β subunits with the tandem α subunit and the other containing two β subunits with the two α_L_ subunits.

The fact that this novel tandem α subunit form has been maintained through the subsequent diversification of three cryptophyte clades suggests that it may have a specific role, perhaps acting as a scaffolding protein that organizes the PBPs in the thylakoid lumen.

## 3 DISCUSSION

The first nuclear genome of a cryptophyte, *Guillardia theta* CCMP2712, showed that while the β subunit of the light harvesting antenna was coded by a single plastid gene, there existed 20 distinct α subunit genes within the nucleus (Curtis et al., 2012). This discovery was in keeping with previous data showing multiple α subunit genes in *Rhodomonas* sp. CS24 (Broughton et al., 2006). Proteomic studies on *G. theta* showed that all α subunit genes resulted in protein products (Kieselbach et al., 2018). The obvious question is why so many α subunits?

Here we have shown that these α subunits control the structural and spectral properties of the mature cryptophyte light harvesting protein. They do this by controlling the quaternary structure (Harrop et al., 2014) and by altering the structural properties of the chromophores and their local environments. Our crystal structures show that within individual PBPs with chemically identical chromophores (“*Chroomonas*” PC645 vs. *Chroomonas* PC630; *H. pacifica* PC577 vs. *H. virescens* PC612), it is the α subunits that dictate both the conformational differences resulting in the rotation of individual pyrrole rings within the chromophores and the chromophore environment. These alterations result in the variation of relative peak heights within the absorption spectra of these PBPs with otherwise identical chromophores (Arpin et al., 2015; Corbella et al., 2019). In contrast, the structures of the individual β subunits are nearly identical, consistent with their high degree of sequence conservation (Apt et al., 1995), while the α subunits show little sequence conservation.

The cryptophyte light harvesting antennas produce a wide variety of spectral features that cover different parts of the visible spectrum (Glazer & Wedemayer, 1995). Primarily, this wide spectral coverage is provided by the array of different linear tetrapyrrole chromophores attached to the PBPs. An individual cryptophyte PBP may carry up to three chemically and spectrally distinct linear tetrapyrroles (such as “*Chroomonas*” PC645 and *Chroomonas* PC630). While closely related PBPs such as *Rhodomonas* PE545 and *C. pyrenoidifera* PE566 vary their spectral properties by replacing DBV by bilin 618 and PEB by bilin 584. The wide variety of PBPs is likely to be present to allow for access to different parts of the visible spectrum depending on the light available.

We have previously shown that the cryptophyte α subunit controls the quaternary structure of the PBP (Harrop et al., 2014). The insertion of an aspartic acid residue in the otherwise strictly conserved chromophore binding motif results in a ~70° rotation of pyrrole ring A in the α19 chromophore (Figures 3a and S2a,b) that alters the quaternary structure from the closed form to the open form where the latter is seen exclusively in *Hemiselmis* PBPs. This structural change alters the spectral properties (Harrop et al., 2014; Arpin et al., 2015).

On examining transcriptome α subunit sequences from *Hemiselmis* species, we discovered that not all sequences contain this aspartic acid single residue insertion that is characteristic of the open form quaternary structure (Figures 7, 8 and S9). While the open form sequence is the most common form in *Hemiselmis* and to date, all crystal structures of *Hemiselmis* PBPs adopt the open quaternary form, characteristic closed form sequences are found in *H. anderseni* (four sequences), *H. virescens* (three sequences), *H. tepida* (four sequences), and *H. rufescens* (four sequences), where these closed form sequences partition into pairs of α_L_ and α_S_ sequences (Figures 8, S9 and Table 2). This suggests that these four *Hemiselmis* species are likely to encode two distinct mature closed form α_L_β.α_S_β complexes.

Our proteomic data on *H. virescens* shows that both open and closed form α subunits are expressed as protein (Figure 7). While it is likely that the major PBP species will be in the open quaternary structure, these data indicate that closed form quaternary structure PBPs are present in these cells.

An even more surprising finding was a single α subunit gene encoding a tandem motif containing two mature α subunit sequences (Figure 9), found in each of four PE species. Using AlphaFold2, we have shown that these tandem α subunit genes predict a structure with two αβ domains connected by a short, flexible linker peptide. This model for the tandem α subunit is reminiscent of the red algal CaRSP proteins, which are scaffolding proteins that contain tandem repeats of a CALM domain (cryptophyte α‐like motif) (Rathbone et al., 2021). In the red algal phycobilisome, the CaRSP scaffolding proteins bind PE β subunits in tandem and anchor them in a linear fashion to the phycobilisome rod structures. It is possible that these cryptophyte tandem α subunits also scaffold linear arrays of β subunits and anchor them to some structures in the thylakoid. We note that the CaRSP proteins do not contain chromophores or chromophore attachment sites, in contrast to these cryptophyte tandem α domain proteins.

Our model for the evolution of the cryptophyte antenna following the endosymbiosis of the red algal progenitor is that one of the smaller red algal CaRSP scaffolding genes survived transfer to the host nucleus, acquired plastid targeting sequence, and was thus able to stabilize the phycobilisome β subunits, which are unstable in isolation (Laos et al., 2017). While other parts of the phycobilisome were gradually being lost, the progenitor cryptophyte α subunit acquired a chromophore binding sequence and with its β subunit became a primitive light‐harvesting antenna, eventually giving rise to the novel cryptophyte antenna family (Rathbone et al., 2021).

The new cryptophyte α subunit underwent multiple rounds of gene duplication and divergence creating the extant multigene families. The complexes formed by these divergent α subunits with conserved β subunits resulted in mature cryptophyte antenna proteins with varied spectral properties. This variation was advantageous in optimizing spectral coverage and antenna structure, with the presence of multiple α subunit genes likely providing the tools for adaptation to altered light conditions.

## 4 MATERIALS AND METHODS

### 4.1 Protein purification for crystallography

*Chroomonas gentoftensis* CCAC1627 and *H. pacifica* CCMP706 were grown in aerated ASP‐H medium (Provasoli et al., 1957; Mcfadden & Melkonian, 1986) at 16°C under a 14/10 h light/dark cycle with light intensities ~50 μmol photons m^−2^ s^−1^. *Cryptomonas pyrenoidifera* CCAP979/61 was grown in freshwater medium Waris‐H (Hoef‐Emden, 2008) at 15°C under a 14/10 h light/dark cycle with light intensities 15–30 μmol photons m^−2^ s^−1^. Cultures were harvested by flow‐through centrifugation and stored at −80°C. Algal cell pellets were thawed, re‐suspended in 2–3 volumes of 25 mM phosphate buffer, pH 7, and homogenized with at Teflon glass homogenizer at 30 rpm. Cells were disrupted in a French press at 1000 psi and centrifuged at 23,000×*g* for 1 h at 4°C. The supernatant was purified via ammonium sulfate cuts (0–50%, 50–60%, 60–70% and 70–80%) by adding solid ammonium sulfate, stirring for 1 h at 4°C and centrifuging at 23,000×*g* for 30 min at 4°C. The 70–80% pellets were resuspended in 25 mM phosphate buffer, pH 7, filtered, dialyzed against the same buffer and loaded onto a Q Sepharose HiLoad 26/10 anion exchange column. The fractions containing the majority of light harvesting protein were selected using the ratio of the appropriate visible absorbance to absorbance at 280 nm and concentrated on a 10 kDa Centriprep (Millipore). The protein was purified by size exclusion chromatography using a Superdex 200 HiLoad 26/60 column. Proteins eluted as a single peak and were concentrated using a 10 kDa cut off Centriprep (Millipore) before snap freezing and storage at −80°C.

### 4.2 Crystallization

The proteins were crystallized using vapor diffusion under the following conditions: PC630—20–28% PEG 3350 HEPES 0.1 M pH 7.5; PC577—15–25% PEG 10k 0.1 M HEPES pH 7.5; and PE566—20–25% PEG 4k, 0.1 M HEPES pH 7.5.

### 4.3 Data collection

Crystals were transferred to cryoprotectant solution of reservoir plus 15% glycerol then flash cooled in liquid nitrogen and mounted in a cryostream for data collection. All final datasets were collected using a ACSD Quantum 315r detector on beamline MX2, Australian Synchrotron (Table S1). Data collection was carried out using Blu‐Ice (McPhillips et al., 2002).

### 4.4 Data reduction and structure determination

All data were processed using XDS (Kabsch, 2010) and SCALA (CCP4; Collaborative Computational Project No 4, 1994). Phasing, auto building and refinement were carried out using PHENIX (Adams et al., 2002). A single β subunit from the structure of PE545 (Wilk et al., 1999) was used as a molecular replacement probe using PHASER (McCoy et al., 2007) as implemented in PHENIX (Adams et al., 2002). Manual adjustments were carried out using COOT (Emsley & Cowtan, 2004). Structural figures were created using PYMOL (DeLano, 2002). Refinement statistics are presented in Table S1.

### 4.5 Structure of PC630

The PC630 structure contains one α_1_β.α_2_β complex in the asymmetric unit. Clear electron density was observed for the complete α_L_ and α_S_ subunits. For each α subunit, Lys4 was modified as 5‐hydroxyl lysine, with the hydroxyl group hydrogen bonded to neighboring water molecules. This modification was previously observed in the *Rhodomonas* sp. CS24 PE545 structure (Wilk et al., 1999). Weak electron density was observed for N‐terminal helix hX (residues Phe5 to Thr10) in the β subunit attached to α_S_. Clear electron density was observed for residues Ala16 (start of the β strand) to the C‐terminal Ala177 in both β subunits. Asn72 is modified as expected to γ‐N‐methyl‐asparagine (Klotz & Glazer, 1987). The only Ramachandran outlier is Thr75 in both β subunits which has a positive *ϕ* angle, as seen in all other cryptophyte and phycobilisome β subunits (Schirmer et al., 1985; Wilk et al., 1999; Harrop et al., 2014).

### 4.6 Structure of PC577

The structure of PC577 contains a single (αβ)_2_ complex in the asymmetric unit. Excellent electron density can be seen for residues: 1–63 and 2–63 for the α subunit; and 4–177 and 5–175 for the β subunit. The only difference between the two αβ protomers is the side chain of Phe30 in the β subunit, which adopts a distinct rotamer due to a slight difference in crystal packing against Asp157 in a symmetry related molecule. Ser75 is a Ramachandran outlier in both β subunits and this feature is common to all β subunit structures determined to date (Schirmer et al., 1985; Wilk et al., 1999; Harrop et al., 2014).

### 4.7 Structure of PE566

The structure of PE566 contains four copies of the (α_L_β).(α_S_β) complex in the asymmetric unit. α_L_ runs from Ala1 to Lys78 while α_S_ goes from Lys1 to Lys70. In the β subunit of the (α_L_β) protomer, density is seen for residues Ala2 to Lys8, which form a distorted helix hX. Clear electron density is observed from Lys16 at the N‐terminus of β strand S1 to Gly178 at the end of helix hH (the C‐terminus). The β subunit in the (α_S_β) protomer starts at Ala15, with no observable density for helix hX. In each β subunit, Asn73 is modified to γ‐N‐methyl‐asparagine, as seen in all closed form cryptophyte PBPs (Klotz & Glazer, 1987). Thr76 is the only Ramachandran outlier as per the other structures.

In the β subunit, there are two discrepancies between the PCR‐derived gene sequence and the observed electron density: Ser44 and Ser66, which correspond to alanine and alanine in the gene sequence, respectively. The electron density of α_L_ corresponds to the PCR‐derived sequence with no alterations. The sequence of α_S_ as seen in the electron density did not match any single known sequence from *C. pyrenoidifera*. Fortunately, the electron density was excellent and could be fit unambiguously (apart from Asp vs. Asn and Glu vs. Gln ambiguities plus some surface lysine residues, where only the first few carbon atoms showed clear density). Comparing this sequence to the transcriptome sequences from *Cryptomonas curvata* CCAP979/52 (MMETSP1050) identified Cry_12417 with 51/71 identical residues, with the strongest identity in the center of the molecule between the conserved Ala8 at the start of β strand S1 and the middle of β strand S2 (Val44). BLAST search using the sequence based on the electron density showed the strongest match to a sequence from *Guiliardia theta* (XP_005828817.1). This sequence shared 49/70 identical residues which included the N‐terminus through β strand S1 and the C‐terminal region from the middle of β strand S2 through the α helix to the C‐terminal loop. Thus, between them, these two sequences account for 63 of the 70 residues in α_S_. The remaining seven residues in α_S_ show unambiguous density, apart from Asp2 and Gln7 which could be asparagine and glutamate, respectively.

### 4.8 Analysis of chromophore geometry

Although largely conjugated, the linear tetrapyrrole chromophores deviate from coplanarity between adjacent pyrrole rings. The central pair of rings (Figure S1, cyan) are, in most cases, coplanar at the resolution of the crystal structures and were not analyzed in detail (some analysis in Figure S6). However, the two outer pyrrole rings (Figure S1, pink) tend to be twisted with respect to the central pair. To measure the twist, the coordinates for each chromophore (including alternate conformers and multiple copies in the asymmetric subunit) were passed into Mathematica and planes were fit to each of the four pyrrole rings. The three atoms defining a bond between each pyrrole ring were also fit to a plane (atoms CH, C_inner_, and C_outer_, green in Figure S1a). The molecular geometry can be described by a pair of dihedral angles (𝜃_inner_, 𝜃_outer_) starting from the central pyrrole (Figure S1a, cyan). Deviations in the bond angle around the bridging carbon atom were not considered (Figure S1a, CH) and assumed it to take consistently 130°. The structure bridging between the outer and inner pyrrole rings comprises two bonds each linking a pyrrole ring (via atoms C_inner_ and C_outer_) to the central bridging atom (CH) as seen in Figure S1a. To define angles between planes which ultimately lead to a dihedral angle pair, normal vectors were found for each ring and bond plane (pink, cyan and green, respectively, Figure S1a), totaling seven normal vectors per chromophore (four pyrrole rings plus three bridging bond planes). The orientation of the normal vectors was chosen based on a flat linear bilin orientation (Figure S1b). Two different approaches were taken based on the type of bond intervening each pair of rings.

If a bond between two rings was not fully conjugated, the angular displacement between the rings, φ, was simply calculated using Equation (1) (where *n*_1_ and *n*_2_ are the unit normal vectors of the rings) and the dihedral approach was ignored. This comparator was used because a more sophisticated analysis using dihedral angles is slightly more geometrically complex and non‐conjugated parts of the chromophore have little bearing on the excitation energy.

(1) $$\varphi =\cos^{-1}\left({\hat{n}}_{1}\cdot {\hat{n}}_{2}\right)$$

If a bond between two rings was fully conjugated, the pair of dihedral angles linking adjacent pyrrole rings were calculated. Two auxiliary vectors (V_inner_ and V_outer_ in Figure S1) were defined for each pair of adjacent pyrrole rings, where vector V_inner_ linked the center of the central pyrrole ring (cyan) to the bridging carbon atom, CH, while vector V_outer_ linked CH to the center of the outer pyrrole ring (pink). The direction of these vectors was defined as always stretching outward from the center of the chromophore (i.e., from the central pyrrole rings, B and C). These auxiliary vectors allow for the definition of a rotation direction (with regards to the right‐hand rule). The dihedral angles, 𝜃 in Figure S1, are thus also found using Equation (1) using the normals for the ring and bond planes instead of just the two rings. The sign of the angular displacement is found by taking the cross product of the normal for each pair (in an order such that they track outward from the center of the chromophore and then the dot product is taken with the V_inner/outer_ vectors). Data were plotted on two‐dimensional dihedral angle plots (𝜃_inner_, 𝜃_outer_). The two dihedral angles are not orthogonal, thus, compensating variations in the two angles can result in an identical *φ* rotation as calculated by single angle φ analysis. To facilitate understanding the relationship between dihedral angles, contours were added in dihedral angle plots to provide an estimate of similarity between angle pairs. The contours are derived (Equation (2)) by taking two rotation matrices, ***R*** (one each to describe the two dihedral angles (𝜃_inner_, 𝜃_outer_)) along their corresponding unit vector V_inner/outer_ (Figure S1) and rotating the unit vector normal to the bond plane (U) and finding the dot product.

(2) $$\varphi =\cos^{-1}\left(R\left({\vec{V}}_{\text{inner}},\theta_{\text{inner}}\right)\vec{U}\cdot R\left({\vec{V}}_{\text{outer}},\theta_{\text{outer}}\right)\vec{U}\right)$$

Assuming the bond angle bridging the two rings is 130°, the equivalent single angle φ can be given by a modified angle sum formula

(3) $$\varphi =\cos^{-1}\left(\cos\left(\theta_{\text{inner}}\right)\cos\left(\theta_{\text{outer}}\right)+\cos\left(130{}^{\circ}\right)\sin\left(\theta_{\text{inner}}\right)\sin\left(\theta_{\text{outer}}\right)\right)$$

where without loss of generality, Equation (3) is derived from Equation (2) using the vectors:

$$U=\left(0,0,01\right)$$

$${\vec{V}}_{\text{outer}}=\left(-\cos\left(130{}^{\circ}\right),\sin\left(130{}^{\circ}\right),0\right)$$

$${\vec{V}}_{\text{inner}}=\left(-1,0,0\right)$$

This can be adjusted for non‐conjugated systems by including an extra rotation to adjust for the tilt on the ring with respect to the bond plane.

Uncertainties were calculated for structures which contained either alternate conformers or contained multiple copies of the mature complex in the asymmetric unit. These are in the form of a standard deviation.

Lastly, alignments in Figure 3 were made using ChimeraX 1.2.5 (Goddard et al., 2018) (align command) by using only the atoms in rings B and C of alignment pairs. Figures for Figure 3 were produced using Pymol 2.5.1 (DeLano, 2002) and angle plots (Figures S2–S6) were produced by Mathematica 12.0.0.0.

### 4.9 Algal cultures for α subunit sequencing

An axenic culture of *Chroomonas mesostigmatica* (CCMP269) and non‐axenic cultures of *Proteomonas sulcata* (CCMP705), *Hemiselmis pacifica* (CCMP706) and *H. cryptochromatica* (CCMP1181) were obtained from the Provasoli–Guillard National Center for Marine Algae and Microbiota (Boothbay Harbor, ME, USA). *P. sulcata* and *C. mesostigmatica* were grown in seawater supplemented with L1 (Guillard & Hargraves, 1993) and K medium nutrients (Keller et al., 1987), respectively. *H. cryptochromatica* and *H. pacifica* were grown in seawater with Prov50 medium nutrients (NCMA medium recipe). Non‐axenic culture of *Cryptomonas pyrenoidifera* (CCAP979/61) was obtained from the Culture Collection of Algae and Protozoa (CCAP, Scotland, UK). *Chroomonas gentoftensis* CCAC1627, *Chroomonas nordstedtii* CCAC1312 and *H. virescens* CCAC1635 were all obtained from Culture Collection of University of Cologne, Germany (all non‐axenic; now Central Collection of Algal Cultures, University Duisburg‐Essen, Germany). CCAC1627 and CCAC1635 were grown in medium ASP‐H; *C. pyrenoidifera* CCAP979/61 and *Chroomonas nordstedtii* CCAC1312 were grown in freshwater medium Waris‐H (Hoef‐Emden, 2008). A non‐axenic culture of *Rhodomonas minuta Skuja* (CPCC344) was obtained from the Canadian Phycological Culture Center and grown in half‐strength CHU‐10 medium. All cultures were grown at 18° and 20 μmol photons m^−2^.s^−1^ light on a 12 h light/12 h dark cycle, if not otherwise specified.

### 4.10 RNA isolation, cDNA synthesis, PCR and sequencing

Cells in exponential phase were harvested and frozen with liquid nitrogen immediately before performing RNA isolation using RNAqueous 4 PCR (Ambion) or Total RNA Isolation Reagent (Advanced Biotechnologies) followed by removal of DNA contamination with DNA‐free™ Kit (Ambion). First strand cDNA was generated using SuperScript® III Reverse Transcriptase (Invitrogen) with random hexamers/nonamers or oligo (dT) as primers and used for degenerate PCR. Degenerate primer pairs for α subunit sequences were based on the available cryptophyte α subunit sequences or on the best partial amino acid sequences derived from electron density maps (*Chroomonas gentoftensis* CCAC1627, *Chroomonas nordstedtii* CCAC1312, *H. virescens* CCAC1635 and *H. pacifica* CCMP706). Degenerate primers for β subunit sequences were designed based on the alignment of DNA sequences from cryptophytes *G. theta* CCMP2712 and *Rhodomonas salina* CCMP1319 and all the available red algal β subunit sequences in Genbank. PCR products were cloned into T‐vectors and isolated colonies were selected randomly for sequencing. The resulting sequences were used to design outward directed PCR primer pairs for cDNA based inverse PCR according to Huang and Chen (Huang & Chen, 2006). All PCR reactions were carried out with Platinum Taq DNA polymerase (Invitrogen). For the complete α subunit sequences, 5′ RACE was done with FirstChoice RLM‐RACE kit (Ambion) or ExactSTART kit (Epicentre). Genomic DNA based inverse PCR was used for the 3′ end of the β subunit. The assembled sequences were confirmed by PCR from the start codon to beyond the stop codon using specific non‐degenerate primers.

### 4.11 Transcriptome analysis

The experimentally determined α subunit sequences were used to search public databases (NCBI or UniProt) and transcriptome data from the Moore Foundation Marine Microbial Eukaryote Transcriptome Sequencing Project (Keeling et al., 2014) using blastP or tblastN. The transcriptome sequences were translated, inspected, and assembled where necessary. Almost all of the deduced protein sequences included at least part of the tripartite targeting sequences. Any sequences that did not have the chromophore‐binding motif were eliminated from consideration.

### 4.12 Comparative sequence analysis

DNA and protein were aligned with MAFFT (Katoh & Standley, 2013) (http://mafft.cbrc.jp/alignment/server/) and refined in BioEdit (Hall, 1999). Protein structure‐based alignment was performed using the online PROMALS3D server (Pei et al., 2008) (http://prodata.swmed.edu/promals3d) with the structure of PDB 1XG0 (Wilk et al., 1999) as constraint.

Tree inferring was implemented by using RAxML‐HPC2 server on XSEDE at Cipres Science Gateway (http://www.phylo.org/portal2/), for protein trees, WAG and GAMMA and Empirical Frequency were chosen as substitution model with 100 rapid bootstrapping runs. Trees were viewed and exported with FigTree v1.4.0.

### 4.13 *Hemiselmis virescens* proteomics survey

*H. virescens* CCAC1635 cells grown under high light (150 μmol photons s^−1^ m^−2^) or low light (40 μmol photons s^−1^ m^−2^) were sedimented by centrifugation at 5000×*g* for 10 min, suspended in a small volume of phosphate‐buffered saline (PBS) and frozen in liquid nitrogen. Upon thawing, the broken cell suspension was centrifuged at 10,000×*g* to remove cell debris, and the supernatant centrifuged at 27,000×*g* to remove thylakoid membrane. Soluble proteins were separated on a 10% Tris‐tricine gel. Following staining with Coomassie G250, the major 8–10 kDa protein band was excised and digested with trypsin, desalted, and subjected to LC–MS/MS following Hippmann et al. (Hippmann et al., 2017). Peak lists were created using Agilent MassHunter software and searched using Mascot (v2.5.1) against the Uniprot_Trembl (v130912) database supplemented with translated sequences from the *H. virescens* transcriptome. Proteins were reported as identified if the species identifier was *H. virescens* with at least 2 different peptides identified with >95% confidence per protein.

### 4.14 Alphafold2 modeling

The model of the *G. theta* phycoerythrin β subunits (NP_050697.1) with the tandem α subunit (XP_005830263.1) phycoerythrin alpha subunit 10 (*G. theta* CCMP2712) was prepared using the default output of 25 models determined by Alphafold2 v2.2.0. Alphafold2 was run from the Deepmind Alphafold2 docker package provided by Github (https://github.com/deepmind/alphafold/releases/tag/v2.2.0) using default settings with a maximum template date cut‐off of 2022‐05‐14, in multimer mode with the default 25 models.

### 4.15 Accession codes

Protein Data Bank: coordinates and structure factors have been deposited under the accession codes: PDB 7T7U (for PC630, from the cryptophyte *Chroomonas gentoftensis* CCAC1627), PDB 7T8S (for PE566, from the cryptophyte *Cryptomonas pyrenoidifera*), and PDB 7T89 (for PC577 from the cryptophyte *H. pacifica* CCMP706).

DNA sequences obtained by PCR and not in Harrop et al. (2014) have been deposited in Genbank under the following accession numbers: *H virescens* (CCAC1635), KC905456‐KC905459; *H. pacifica* (CCMP706), KF254463, KF254473‐KF254575; *H. cryptochromatica* (CCMP1181), TBA; “*Chroomonas*” *mesostigmatica* (CCMP 269), KF976873‐7; *Chroomonas* spp. (CCAC1627 and CCAC1312), KF254461, 462, 464–472; *Proteomonas sulcata* (CCMP705), KF976867‐976872; *Rhodomonas minuta* (CPCC344), KF976861‐976866; *Cryptomonas pyrenoidifera* (CCAP979/61) TBA.

## AUTHOR CONTRIBUTIONS

**Katharine A Michie:** Formal analysis (equal); investigation (equal); validation (equal); writing – original draft (supporting); writing – review and editing (supporting). **Stephen J Harrop:** Formal analysis (equal); investigation (equal); methodology (equal); validation (supporting); writing – review and editing (supporting). **Harry W Rathbone:** Formal analysis (supporting); investigation (supporting); methodology (supporting); validation (supporting); writing – original draft (supporting); writing – review and editing (supporting). **Krystyna E Wilk:** Formal analysis (supporting); investigation (supporting); methodology (supporting). **Chang Y Teng:** Formal analysis (supporting); investigation (supporting); methodology (supporting). **Kerstin Hoef‐Emden:** Formal analysis (supporting); investigation (supporting); methodology (supporting); writing – review and editing (supporting). **Roger G Hiller:** Conceptualization (supporting); formal analysis (supporting); investigation (supporting); methodology (supporting); validation (supporting); writing – review and editing (supporting). **Beverley R Green:** Conceptualization (supporting); formal analysis (supporting); investigation (supporting); methodology (supporting); resources (supporting); validation (supporting); writing – original draft (supporting); writing – review and editing (supporting). **Paul M. Curmi:** Conceptualization (lead); formal analysis (supporting); funding acquisition (lead); investigation (equal); methodology (supporting); project administration (lead); resources (lead); supervision (lead); validation (supporting); writing – original draft (lead); writing – review and editing (lead).

## AUTHOR CONTRIBUTIONS

Kerstin Hoef‐Emden and Chang Ying Teng cultured *Chroomonas gentoftensis* CCAC1627 B, *H. virescens* CCAC1635 B, *H. pacifica* CCMP706 and *Cryptomonas pyrenoidifera* CCAP979/61. Krystyna E. Wilk purified and crystallized all proteins. Stephen J. Harrop collected and reduced all diffraction data. Stephen J. Harrop, Katharine A. Michie, Harry W. Rathbone, and Paul M. G. Curmi determined and refined all crystal structures. All sequencing and sequence analysis was carried out by Chang Ying Teng, Beverley R. Green, and Roger G. Hiller. All authors participated in interpreting the results and writing the manuscript.

## CONFLICT OF INTEREST STATEMENT

The authors declare no competing interests.

## Supporting information


**Appendix S1:** Supporting Information

## Data Availability

The data that support the findings of this study are openly available in the PDB under accession codes: 7T7U, 7T8S and 7T89.
