# Supplementary material for: Molecular structures reveal the origin of spectral variation in cryptophyte light harvesting antenna proteins
Source: Protein Sci. 2023 Feb 24;32(3):e4586. doi: 10.1002/pro.4586 (PMC9951199; doi:10.1002/pro.4586)
Supplement: Supplementary file 1 — Appendix S1: Supporting Information [file PRO-32-e4586-s001.pdf]

# Molecular structures reveal the origin of spectral variation in cryptophyte light harvesting antenna proteins

Katharine A. Michie[1,2,3†] Stephen J. Harrop[1,4†], Harry W. Rathbone[1,2], Krystyna E. Wilk[1], Chang Ying Teng[5], Kerstin Hoef-Emden[6], Roger G. Hiller[7], Beverley R. Green[5] & Paul M. G. Curmi[1,2*]

**Supplementary material**

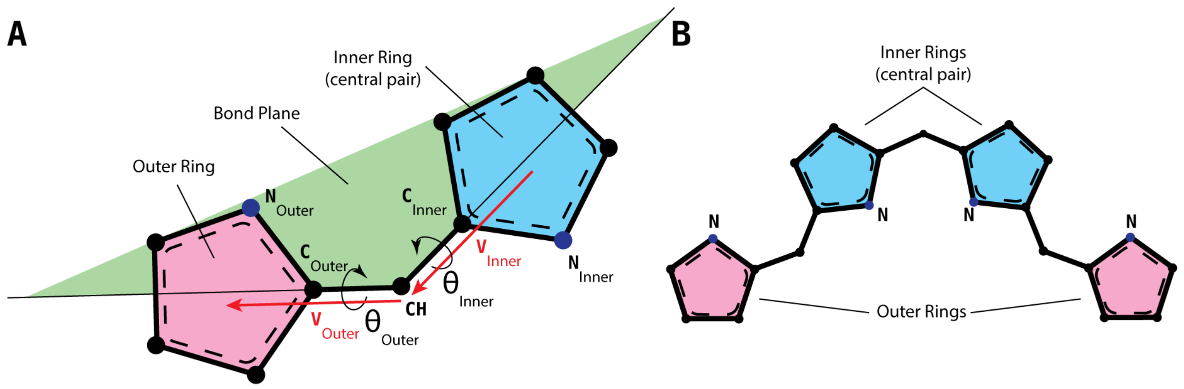


**Figure S1**

***Chromophore torsion angle geometry.*** **A.** shows the geometric construction for determining the two dihedral angles ($\Theta_{inner}$, $\Theta_{outer}$) that link the inner (blue) pyrrole ring (central pair) to the outer (pink) pyrrole rings. Key atoms are labelled, and the bond plane is shown in green with its normal vector (and also those of the pyrrole rings) pointing out of the page. Auxiliary vectors $V_{inner}$ and $V_{outer}$ are also defined in red. **B.** shows the layout of the tetrapyrrole chromophore with nitrogen atoms marked.

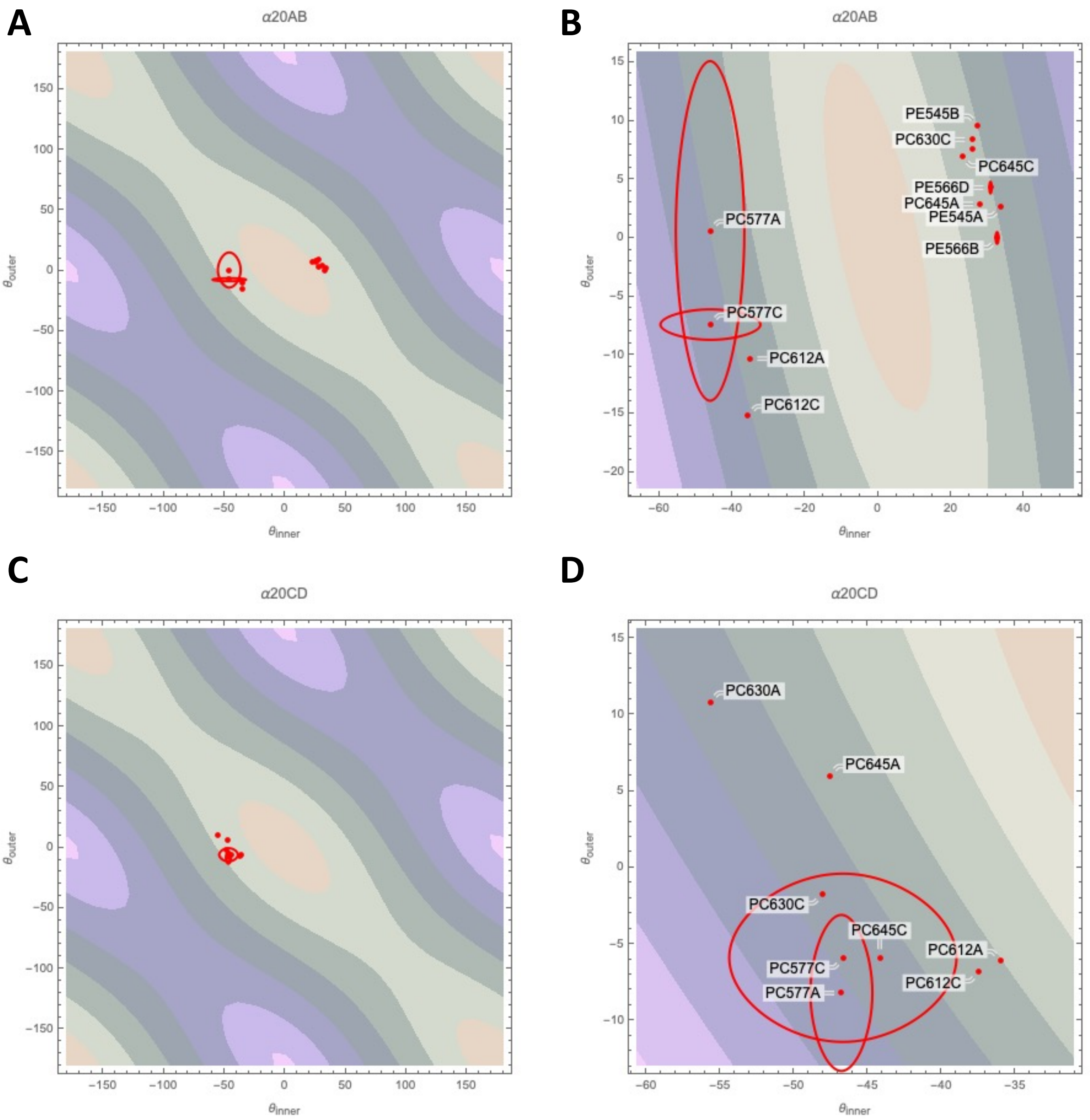


**Figure S2**

***Chromophore torsion angles for α chromophore.*** **A**. torsion angle between pyrrole rings A and B. **B**. Expansion of the central region in **A**. **C**. torsion angles between pyrrole rings C and D. **D**. Expansion of the central region in **C**. PC630A and PC645A in **D** correspond to closed form $\alpha_L$ chromophores (compared to PC630C and PC645C representing $\alpha_S$ chromophores). PC612 can also be compared to PC577 in **D** and in **B**. Uncertainty ellipses are provided for PC577A and PC577C which have alternative chromophore conformers. Numerical values for angles presented are given in **Table S2**.

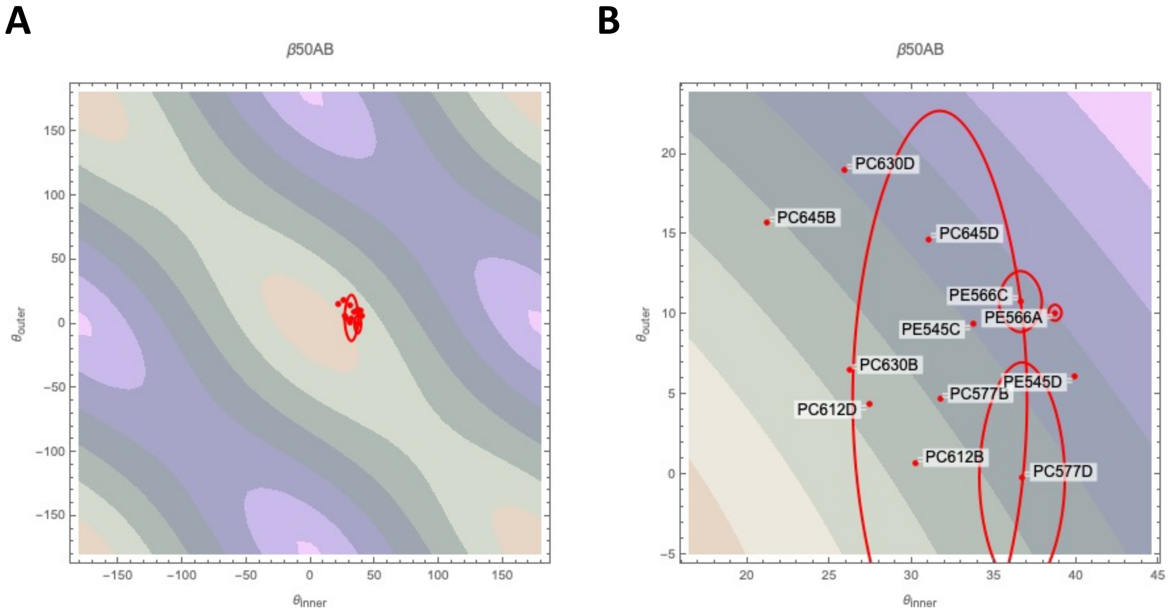


**Figure S3**

***Chromophore torsion angles for β50/61 chromophore.*** **A**. torsion angle between pyrrole rings A and B. **B**. Expansion of the central region in **A**. Torsion angles between pyrrole rings C and D are not presented since they are not linked in a planar fashion by sp2 carbon atoms. The most prominent difference here is the separation in angle between closed form chains within either PC645 or PC630, where a chiral adjustment is made in the thioether bond to the Cys50 within the protein (Figure S7). Uncertainty ellipses are provided for proteins with either multiple copies in the ASU or alternative conformers. Numerical values for angles presented are given in **Table S3**.

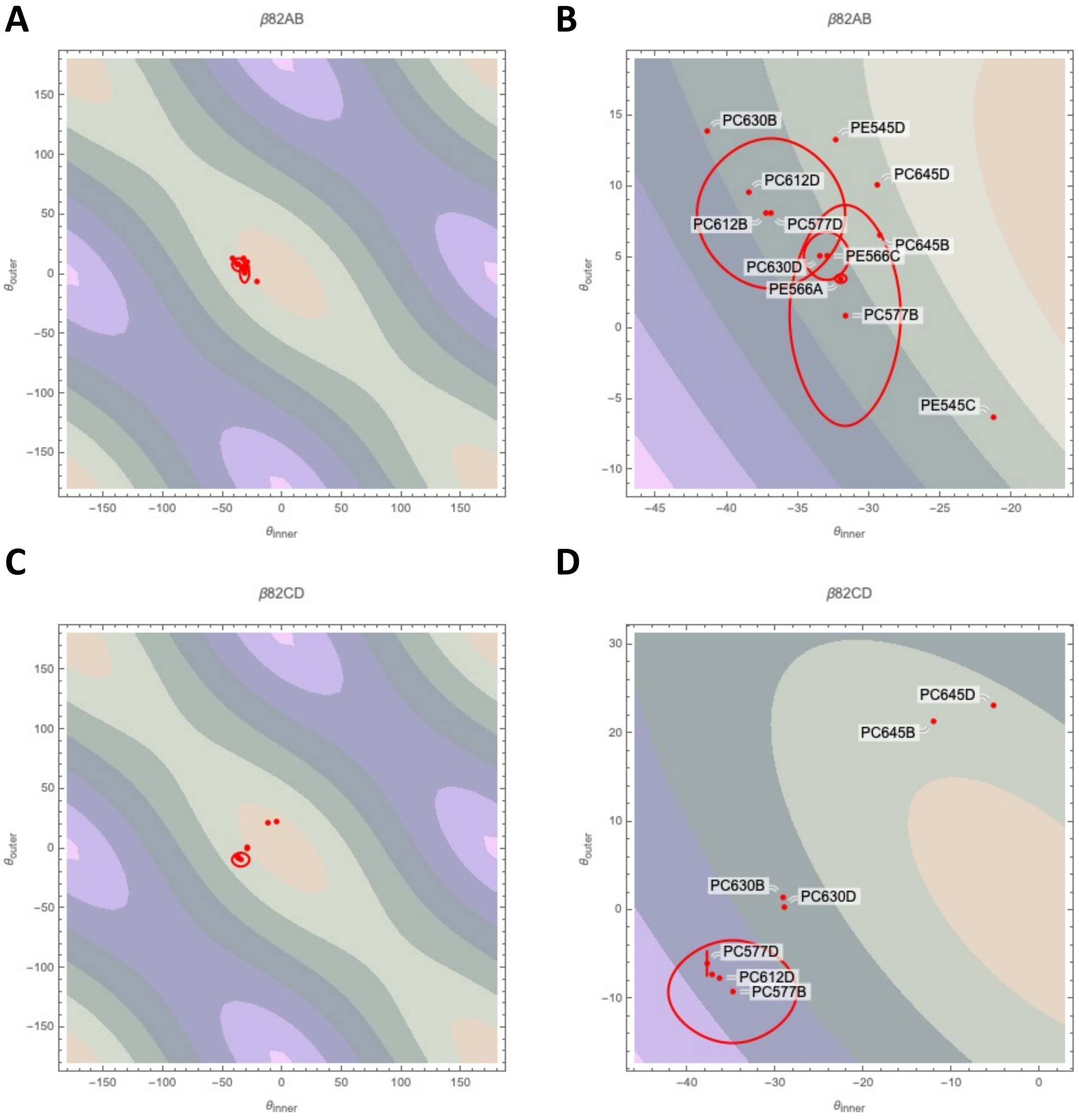


**Figure S4**

***Chromophore torsion angles for β82 chromophore. A***. torsion angle between pyrrole rings A and B. **B**. Expansion of the central region in **A**. **C**. torsion angles between pyrrole rings C and D. **D**. Expansion of the central region in **C**. The most notable difference here is correlated to the identity of the residue preceding the conserved K/RAP of the CALM motif: Leu, Gln or Ser for PC645, PC630 and PC577/PC612 clusters, respectively in **D**. Uncertainty ellipses are provided for proteins with either multiple copies in the ASU or alternative conformers. Numerical values for angles presented are given in **Table S4**.

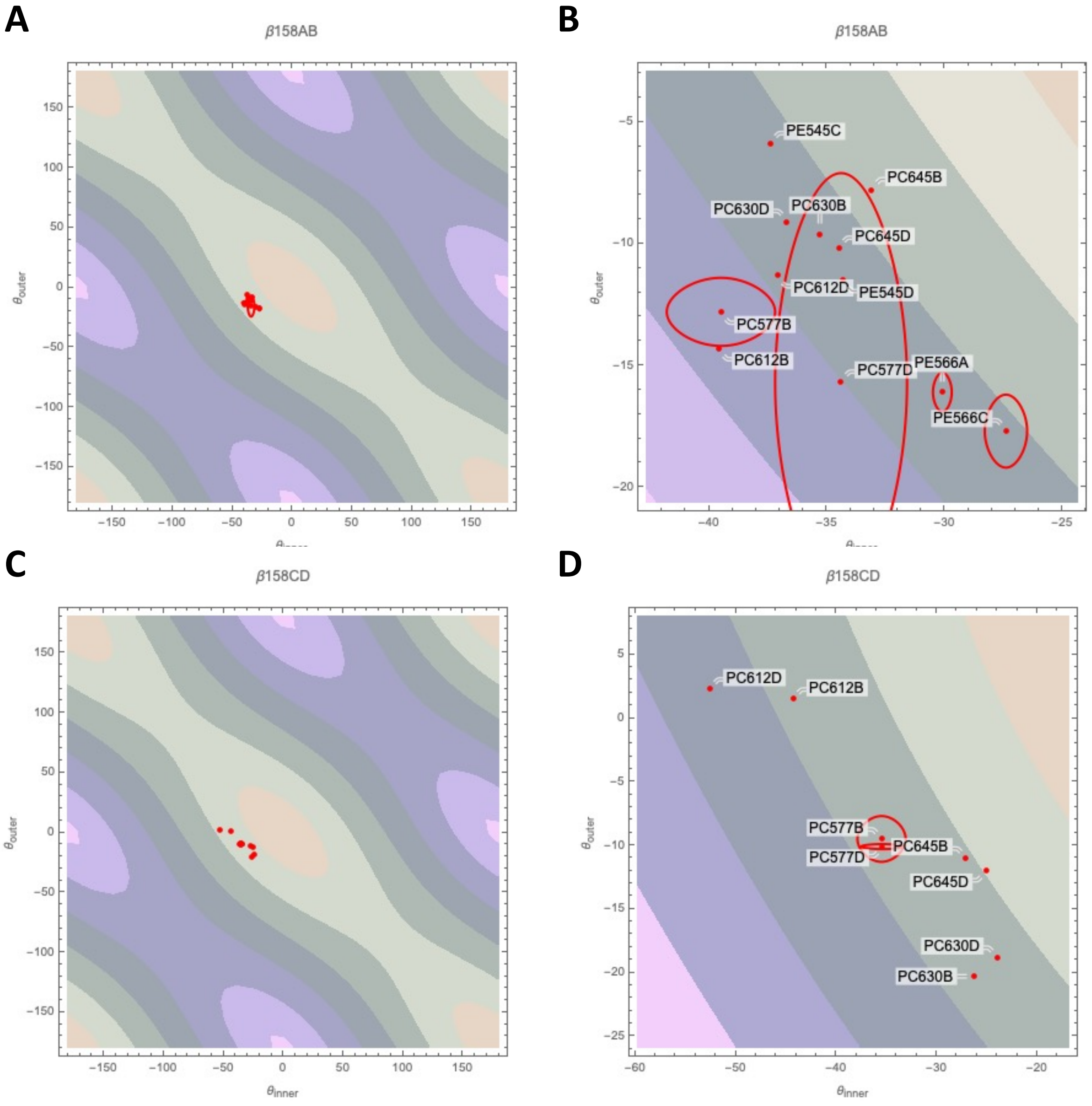


**Figure S5**

***Chromophore torsion angles for β158 chromophore.*** **A**. torsion angle between pyrrole rings A and B. **B**. Expansion of the central region in **A**. **C**. torsion angles between pyrrole rings C and D. **D**. Expansion of the central region in **C**. The most notable difference is between PC577 and PC612 **D**. Uncertainty ellipses are provided for proteins with either multiple copies in the ASU or alternative conformers. Numerical values for angles presented are given in **Table S5**.

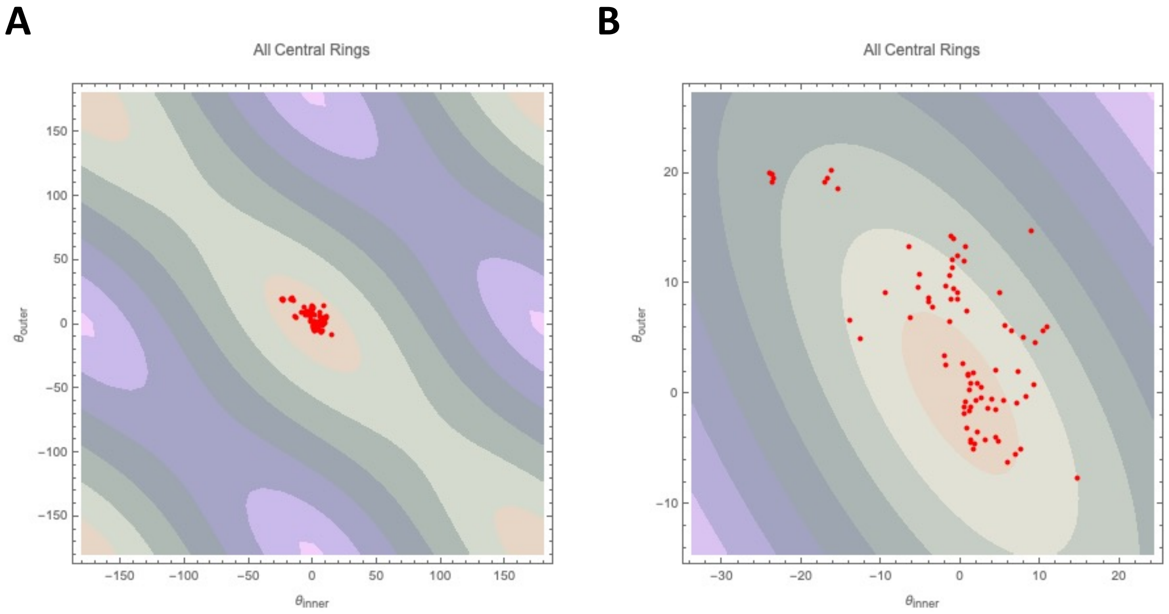


**Figure S6**

***Chromophore torsion angles between the central pyrrole rings for all chromophores.*** **A**. torsion angle between central pyrrole rings B and C. **B**. Expansion of the central region in **A**. This shows a tightly planar geometry for the central pair.

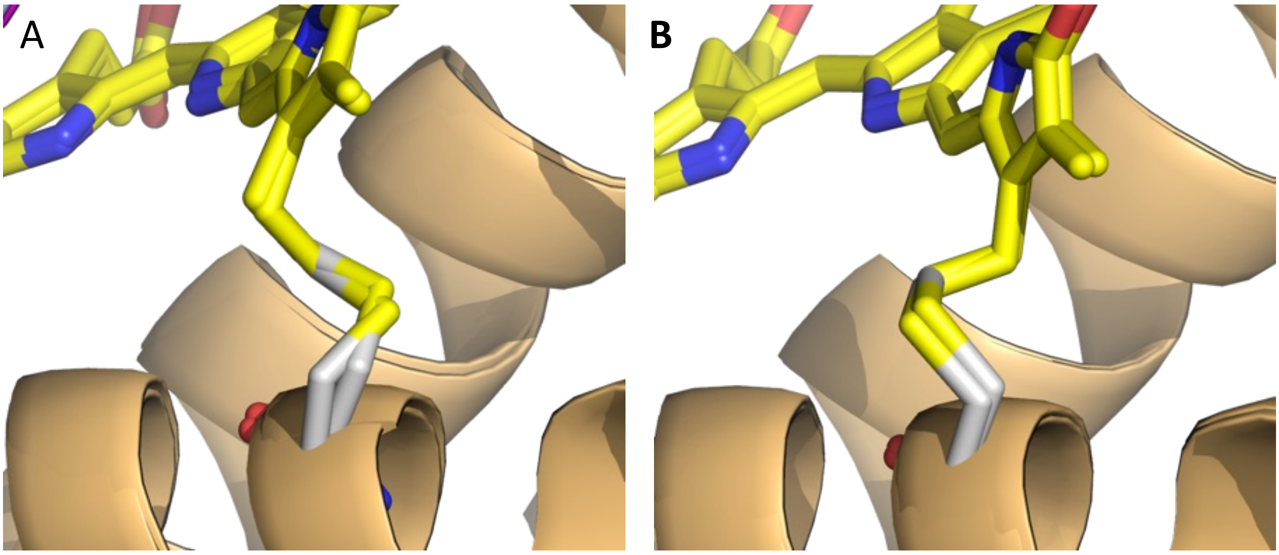


**Figure S7**

***Protomers $\alpha_L\beta$ and $\alpha_S\beta$ show asymmetry in the chirality of the thioether linkage between Cys50 and pyrrole ring A of chromophore β50/61 in both PC645 and PC630.***
**A**. $\alpha_L\beta$ shows a right handed thioether linkage while **B**. $\alpha_S\beta$ shows a left handed thioether linkage in both PC645 and PC630.

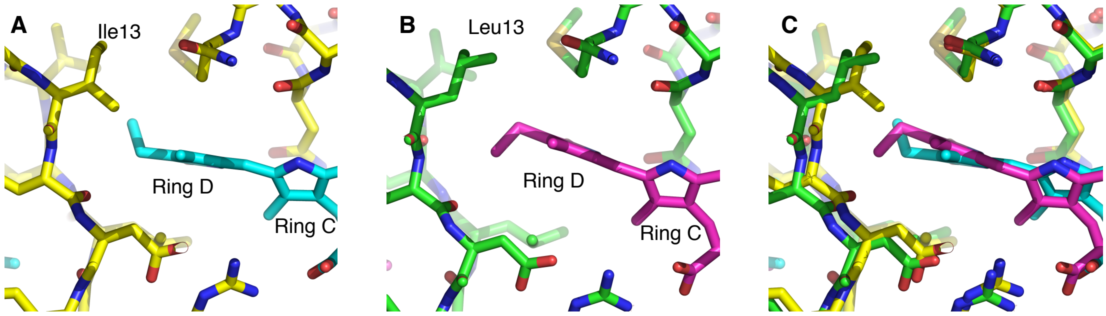


**Figure S8**

***Rotation of pyrrole ring D on β158 chromophore*.** **A.** PC577 pyrrole ring D (seen edge on) is nested against the β sheet formed by the α subunit (strand S1 foreground, strand S2 rear). **B**. there is a rotation of (+13±6,-12±1) relative to pyrrole ring C (seen face on) in PC612 compared to PC577 due to a shift in the α subunit. **C.** the overlay of the two structures based on the β subunit.

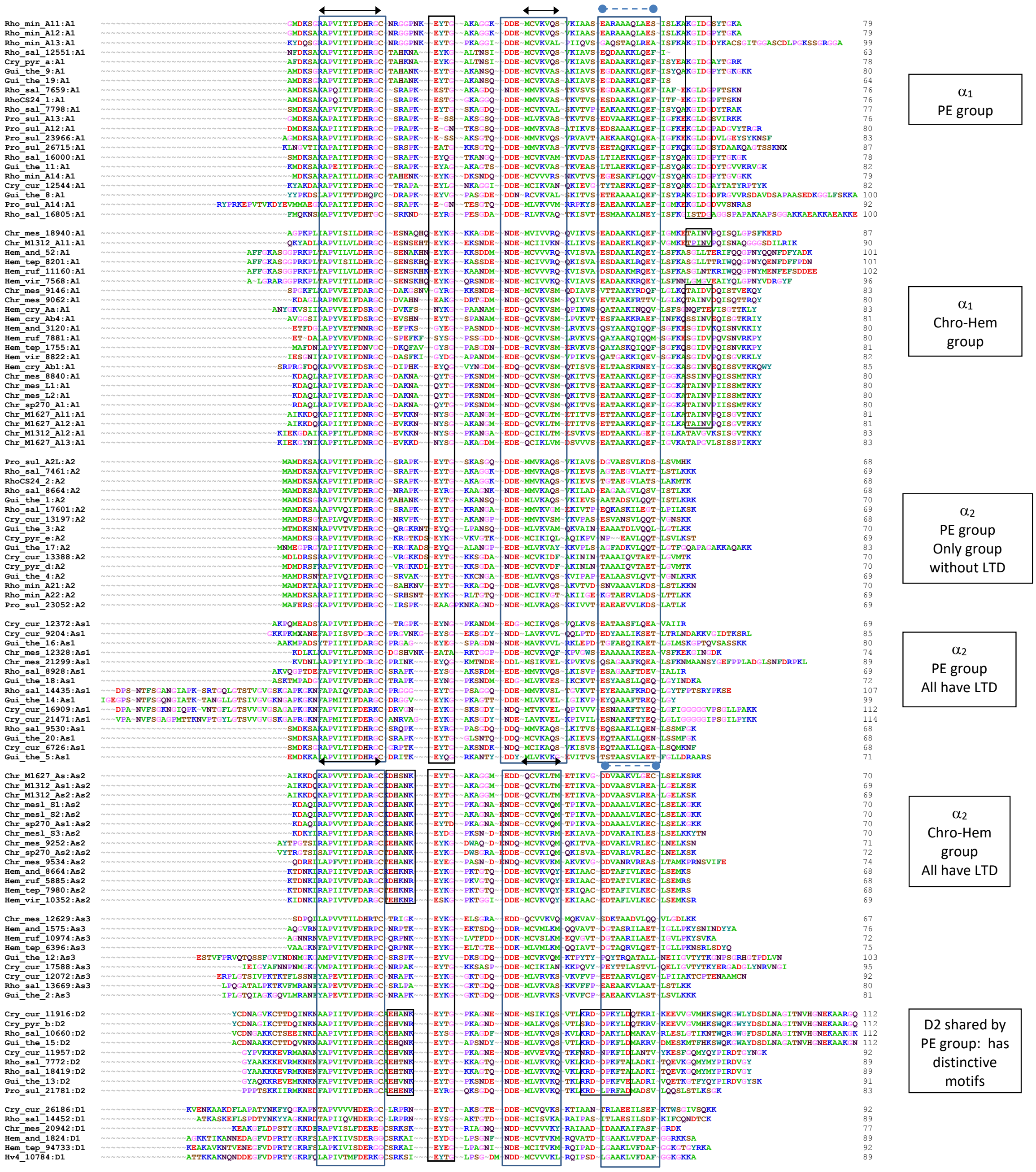


## Figure S9

***Alignment of all available mature, closed form α subunit sequences.*** Key: β sheet: (S1, S2) solid double arrows; helix: dotted line with ball ends; boxes delineate conserved motifs; “PE group” = Hoef-Emden (1) Clades 2, 3, 4, 5 all with PE but not PC.

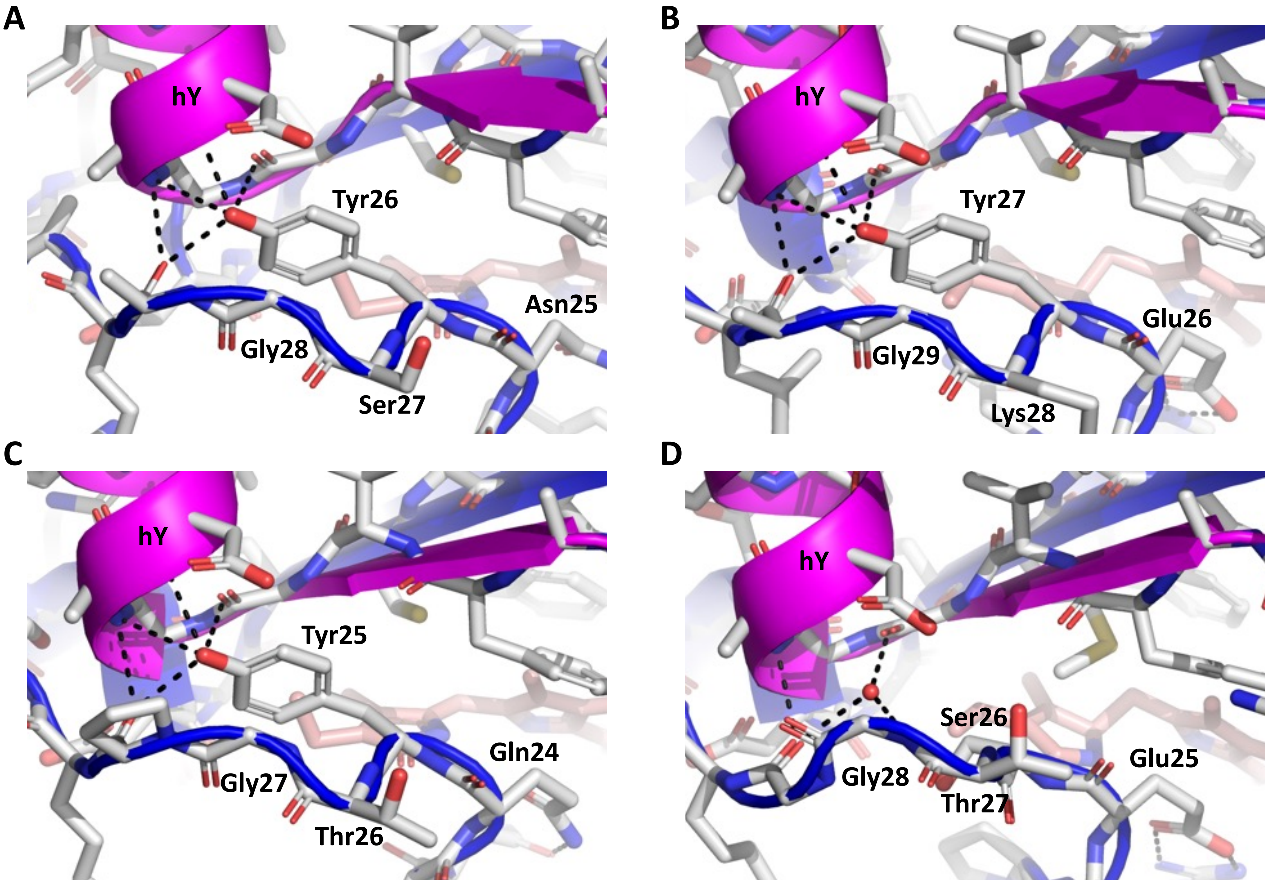


**Figure S10**

***The α subunit EYxG motif shown for $\alpha_L$ subunits.*** **A**. PC630: the motif is NYSG. Tyr26 OH acts as an N-cap to β subunit helix hY. **B**. PE566: the motif is EYKG. Tyr27 OH acts as an N-cap to β subunit helix hY. Glu26 forms a salt bridge with a preceding His (bottom right corner). **C**. PC645: the motif is QYTG. Tyr25 OH acts as an N-cap to β subunit helix hY. Gln24 forms a hydrogen bond with a preceding Asn (bottom right corner). **D**. PE545: the motif is ESTG. A water molecule acts as the N-cap to β subunit helix hY. Glu25 forms a salt bridge with a preceding Arg (bottom right corner).

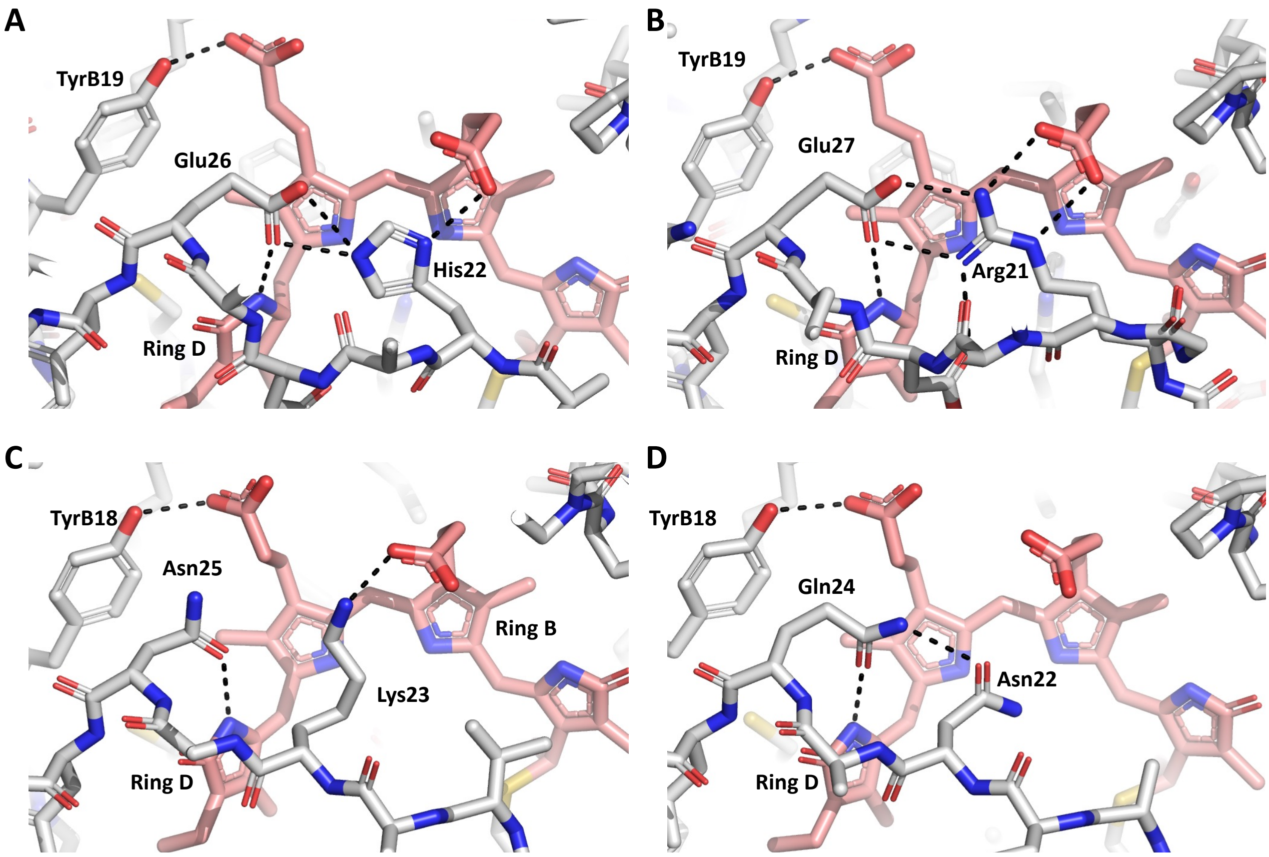


**Figure S11**

***Salt bridge/hydrogen bonding network stabilises the conformation of the α chromophore.*** **A**. PE566 $\alpha_L$ chromophore loop showing His-Glu salt bridge. Structurally homologous His-Glu salt bridges are observed in $\alpha_S$ chromophore loops of PC645 and PC630. **B**. PE566 $\alpha_S$ chromophore loop showing Arg-Glu salt bridge. Structurally homologous Arg-Glu salt bridges are observed in chromophore loops of PE545 $\alpha_L$ and $\alpha_S$. **C**. Chromophore loop of PC630 $\alpha_L$ where the Glu of the EYxG motif is replaced by Asn25 and Lys23 makes a salt bridge to the chromophore propionate attached to pyrrole ring B. **D**. Chromophore loop of PC645 $\alpha_L$ where the Glu of the EYxG motif is replaced by Gln24 which makes a hydrogen bond to Asn22. Note: Tyr18 from the β subunit makes a hydrogen bond to the propionate attached to pyrrole ring C of the α chromophore (Tyr19 in PE566).

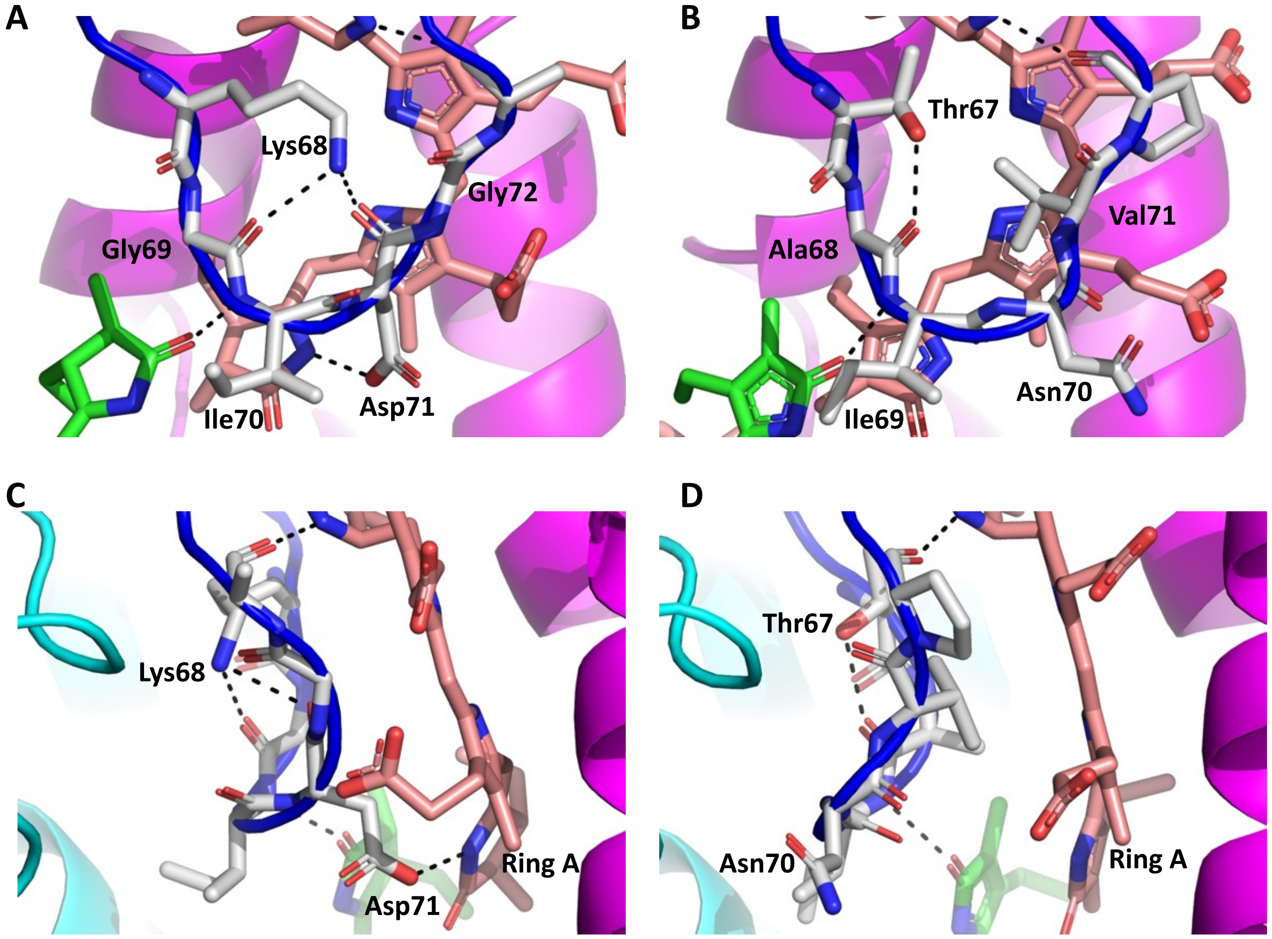


**Figure S12**

***$\alpha_L$ C-terminal loop motif covers the β50/61 chromophore.*** **A**. and **C**. the KGIDG motif of PE566 at the apex of the C-terminal loop. Lys68 makes hydrogen bonds to the carbonyl groups of Gly69 and Asp71. **B**. and **D**. the TAINV motif of PC630 at the apex of the C-terminal loop. Thr67 is only able to hydrogen bond to the carbonyl group of Ala68. Panels **C**. and **D**. are rotated by -90° compared to panels **A**. and **B**. **C**. shows Asp71 making a hydrogen bond to the nitrogen atom of pyrrole ring A in the β50/61 chromophore. **D**. in contrast, Asn70 points away from the β50/61 chromophore.

***Table S1    Data reduction & refinement statistics***

| | PC630 | PC577 | PE566 |
|---|---|---|---|
| PDB code | 7T7U | 7T89 | 7T8S |
| **Data collection**[a] | | | |
| Wavelength | 0.95369Å | 0.953698Å | 1.2632Å |
| Resolution range | 39.5-1.8 (1.84-1.8)* | 22.28-1.0 (1.036-1.0)* | 26.77-2.0 (2.072-2.0)* |
| Space group | I 2 2 2 | P 21 21 21 | P 41 21 2 |
| Unit cell (**a**, **b**, **c** in Å; α, β, γ in °) | 90.2941 93.4114 132.024 90 90 90 | 42.1161 95.3221 125.577 90 90 90 | 178.359 178.359 173.546 90 90 90 |
| Total reflections | 557,688 (51,481) | 2,744,443 (119,527) | 374,545 (37,074) |
| Unique reflections | 51,261 (2,943) | 254,439 (17,139) | 187,274 (18,537) |
| Multiplicity | 4.3 (4.0) | 10.8 (6.9) | 2.0 (2.0) |
| Completeness (%) | 98.8 (96.3) | 93.4 (60.1) | 99.78 (100.00) |
| Mean I/sigma(I) | 8.5 (1.5) | 13.1 (1.7) | 6.18 (1.04) |
| Wilson B-factor | 16.01 | 8.69 | 27.00 |
| R-merge | 0.096 (0.658) | 0.061 (0.411) | 0.056 (0.550) |
| R-meas | 0.123 (0.844) | 0.078 (0.556) | 0.080 (0.778) |
| R-pim | 0.076 (0.522) | 0.049 (0.372) | 0.056 (0.550) |
| CC1/2 | 0.995 (0.670) | 0.993 (0.720) | 0.997 (0.645) |
| **Refinement**[b] | | | |
| Reflections used in refinement | 51,252 (4,939)[†] | 253,237 (17,139) | 186,968 (18,537) |
| Reflections used for R-free | 2,622 (254) | 12,707 (884) | 9,630 (954) |
| R-work | 0.1577 (0.2283) | 0.1326 (0.2496) | 0.2004 (0.3230) |
| R-free | 0.1849 (0.2447) | 0.1427 (0.2543) | 0.2353 (0.3430) |
| CC(work) | 0.582 (0.646) | 0.577 (0.707) | 0.966 (0.807) |
| CC(free) | 0.608 (0.651) | 0.510 (0.695) | 0.927 (0.747) |
| Number of non-hydrogen atoms | 4,482 | 5,540 | 17,083 |
| macromolecules | 3,680 | 4,091 | 14,408 |
| ligands | 648 | 1,296 | 2,630 |
| solvent | 452 | 761 | 1,245 |
| Protein residues | 481 | 470 | 1,933 |
| RMS(bonds) | 0.012 | 0.006 | 0.003 |
| RMS(angles) | 1.01 | 0.91 | 0.56 |

| | | | |
|---|---|---|---|
| Ramachandran favored (%) | 97.82 | 98.70 | 98.66 |
| Ramachandran allowed (%) | 1.96 | 1.30 | 1.34 |
| Ramachandran outliers (%) | 0.22 | 0.00 | 0.00 |
| Rotamer outliers (%) | 0.74 | 0.44 | 0.26 |
| Clashscore | 1.90 | 0.89 | 1.04 |
| Average B-factor | 18.01 | 12.47 | 34.75 |
| macromolecules | 17.70 | 11.73 | 35.21 |
| ligands | 16.27 | 8.99 | 31.35 |
| solvent | 21.89 | 19.60 | 33.45 |
| Number of TLS groups | 17 | | 84 |

*Values in parentheses are for the highest resolution shell

[a]Calculated by AIMLESS (CCP4 (2)) & PHENIX (3)

[b]Calculated by PHENIX (3)

[†]Note: Highest resolution shell used in refinement of PC630 was larger: 1.864-1.80Å

***Table S2*** *Chromophore torsion angles for the α chromophore. Standard deviation is over multiple conformers or copies in the ASU.*

| Protein | Bilin | Chain | A-B (Inner, Outer) | C-D (Single OR Inner, Outer) |
|---|---|---|---|---|
| PE545 | DBV | A | (33.6, 2.7) | 44.9 |
| PE545 | DBV | B | (27.2, 9.6) | 47.4 |
| PE566 | CB8 | B | (32.6 ± 0.4, 0 ± 0.5) | 43.2 ± 8.6 |
| PE566 | CB8 | D | (30.8 ± 0.3, 4.3 ± 0.5) | 44.1 ± 1.5 |
| PC577 | PCB | A | (-46.1 ± 9.4, 0.6 ± 14.5) | (-46.8 ± 2.1, -8.2 ± 5.1) |
| PC577 | PCB | C | (-46 ± 13.7, -7.4 ± 1.3) | (-46.7 ± 7.7, -5.9 ± 5.5) |
| PC612 | PCB | A | (-35.1, -10.3) | (-36, -6.1) |
| PC612 | PCB | C | (-35.7, -15.2) | (-37.5, -6.8) |
| PC630 | M1V | A | (25.8, 7.6) | (-55.7, 10.8) |
| PC630 | M1V | C | (25.7, 8.4) | (-48.1, -1.7) |
| PC645 | M1V | A | (28, 2.9) | (-47.6, 6) |
| PC645 | M1V | C | (23, 7) | (-44.2, -5.9) |

***Table S3*** *Chromophore torsion angles for the β50/61 chromophore. Standard deviation is over multiple conformers or copies in the ASU.*

| Protein | Bilin | Chain | A-B (Inner, Outer) | C-D (Single) |
|---|---|---|---|---|
| PE545 | PEB | C | (33.7, 9.4) | 31.4 |
| PE545 | PEB | D | (39.9, 6.1) | 27.1 |
| PE566 | DB4 | A | (38.7 ± 0.4, 10.1 ± 0.5) | 31.8 ± 0.5 |
| PE566 | DB4 | C | (36.6 ± 1.3, 10.8 ± 1.9) | 31.2 ± 1.5 |
| PC577 | DBV | B | (31.7 ± 5.3, 4.7 ± 18) | 29.4 ± 1.8 |
| PC577 | DBV | D | (36.7 ± 2.6, -0.2 ± 7.2) | 31.4 ± 6 |
| PC612 | DBV | B | (30.2, 0.7) | 31.5 |
| PC612 | DBV | D | (27.4, 4.4) | 29.6 |
| PC630 | DBV | B | (26.2, 6.5) | 32.6 |
| PC630 | DBV | D | (25.9, 19) | 27.8 |
| PC645 | DBV | B | (21.2, 15.7) | 32.7 |
| PC645 | DBV | D | (31, 14.7) | 28.9 |

***Table S4*** *Chromophore torsion angles for β82 chromophore. Standard deviation is over multiple conformers or copies in the ASU.*

| Protein | Bilin | Chain | A-B (Inner, Outer) | C-D (Single OR Inner, Outer) |
|---|---|---|---|---|
| PE545 | PEB | C | (-21.3, -6.3) | 27.5 |
| PE545 | PEB | D | (-32.4, 13.3) | 27.7 |
| PE566 | PEB | A | (-32 ± 0.4, 3.5 ± 0.3) | 26.5 ± 1.4 |
| PE566 | PEB | C | (-33 ± 1.6, 5.1 ± 1.7) | 28.3 ± 0.8 |
| PC577 | PCB | B | (-31.7 ± 3.9, 0.9 ± 7.8) | (-34.9 ± 7.3, -9.2 ± 5.8) |
| PC577 | PCB | D | (-36.9 ± 5.2, 8.1 ± 5.3) | (-37.8 ± 0.0, -6 ± 1.5) |
| PC612 | PCB | B | (-37.3, 8.1) | (-37.2, -7.3) |
| PC612 | PCB | D | (-38.5, 9.6) | (-36.4, -7.6) |
| PC630 | PCB | B | (-41.4, 13.9) | (-29.2, 1.5) |
| PC630 | PCB | D | (-33.5, 5.1) | (-29, 0.3) |
| PC645 | PCB | B | (-29.3, 6.6) | (-12.1, 21.3) |
| PC645 | PCB | D | (-29.4, 10.1) | (-5.3, 23.1) |

***Table S5*** *Chromophore torsion angles for β158 chromophores. Standard deviation is over multiple conformers or copies in the ASU.*

| Protein | Bilin | Chain | A-B (Inner, Outer) | C-D (Single OR Inner, Outer) |
|---|---|---|---|---|
| PE545 | PEB | C | (-37.4, -5.9) | 33 |
| PE545 | PEB | D | (-34.3, -11.5) | 31.9 |
| PE566 | CB4 | A | (-30.1 ± 0.4, -16.1 ± 0.8) | 33.2 ± 1 |
| PE566 | CB4 | C | (-27.4 ± 0.9, -17.7 ± 1.5) | 31.7 ± 0.9 |
| PC577 | PCB | B | (-39.5 ± 2.3, -12.8 ± 1.4) | (-35.5 ± 2.4, -9.5 ± 1.8) |
| PC577 | PCB | D | (-34.4 ± 2.8, -15.7 ± 8.6) | (-35.4 ± 2.1, -10.1 ± 0.2) |
| PC612 | PCB | B | (-39.6, -14.3) | (-44.3, 1.5) |
| PC612 | PCB | D | (-37.1, -11.3) | (-52.6, 2.3) |
| PC630 | PCB | B | (-35.3, -9.6) | (-26.3, -20.3) |
| PC630 | PCB | D | (-36.7, -9.1) | (-24, -18.8) |
| PC645 | PCB | B | (-33.1, -7.8) | (-27.1, -11) |
| PC645 | PCB | D | (-34.5, -10.2) | (-25.1, -12) |

**References**


1. Hoef-Emden, K. (2008) Molecular phylogeny of phycocyanin-containing cryptophytes: Evolution of biliproteins and geographical distribution. Journal of Phycology 44, 985-993

2. Collaborative Computational Project No 4 (1994) The CCP4 suite: programs for protein crystallography. Acta Cryst. D 50, 760-763

3. Adams, P. D., Grosse-Kunstleve, R. W., Hung, L. W., Ioerger, T. R., McCoy, A. J., Moriarty, N. W., Read, R. J., Sacchettini, J. C., Sauter, N. K., and Terwilliger, T. C. (2002) PHENIX: building new software for automated crystallographic structure determination. Acta Crystallogr D Biol Crystallogr 58, 1948-1954
